# How Proton Incorporation Reshapes Lattice Dynamics In BaSnO_3_‐Type Proton Conductors

**DOI:** 10.1002/advs.76065

**Published:** 2026-06-15

**Authors:** Artur Braun, Alexey Rulev, Nobumoto Nagasawa, Hongxin Wang, Tatyana Bendikov, Vladimir Pomjakushin, Martin Kunz, Yoshitaka Yoda, Qianli Chen, Stephen P. Cramer

**Affiliations:** ^1^ Laboratory for High Performance Ceramics Empa Swiss Federal Institutes of Technology Dübendorf Switzerland; ^2^ Precision Spectroscopy Division Hyogo Japan; ^3^ SETI Institute Mountain View USA; ^4^ Department of Chemical Research Support Weizmann Institute of Science Rehovot Israel; ^5^ Laboratory for Neutron Scattering and Imaging Paul Scherrer Institute Villigen PSI Switzerland; ^6^ Advanced Light Source Ernest Orlando Lawrence Berkeley National Laboratory Berkeley USA; ^7^ Global College Shanghai Jiao Tong University Shanghai China

**Keywords:** lattice dynamics, NRVS, phonon dos, proton conductor, proton–phonon coupling

## Abstract

Proton conduction in acceptor‐doped perovskites is fundamentally a vibronic process: mobile $H^{+}$ and $D^{+}$ do not move independently, but dynamically co‐vibrate with the surrounding oxygen–metal framework. Direct experimental evidence for this behavior is presented using in situ 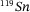 nuclear resonance vibrational spectroscopy (NRVS) on hydrated, deuterated, and dry $BaSn_{1-x}Y_{x}O_{3-\delta }$. Hydration induces systematic redistributions in the Sn‐projected phonon density of states (PDOS), including an upshift of the first spectral moment by about 0.4 meV, indicating a stiffening of the extended Sn–O network.

H/D isotopic substitution leaves the Sn‐projected PDOS largely unchanged, with only subtle isotope‐dependent spectral reweighting, demonstrating that protonic degrees of freedom are not localized oscillators but are embedded in collective lattice modes. These results are rationalized using a classical coupled proton–phonon oscillator model that links the observed PDOS variations to changes in effective force constants and vibrational mass terms. The model captures how $H^{+}$ and $D^{+}$ participate in cooperative lattice dynamics rather than forming isolated OH/OD entities.

Overall, NRVS probes proton–lattice coupling in ceramic proton conductors and quantitatively describes how protonic defects modulate host lattice dynamics to enable phonon‐assisted long‐range proton transport.

## Graphical abstract

Yttrium‐doped BaSnO3 exhibits isotope‐dependent changes in its low‐energy vibrational density of states upon hydration. Comparison of dry, H2O‐, and D2O‐treated samples re‐veals mass‐dependent phonon renormalization linked to proton dynamics near oxygen va‐cancies, providing experimental insight into hydrogen‐coupled lattice excitations in proton conducting perovskite oxides.

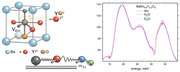

## 1 Introduction

Proton conduction in inorganic solids is a cooperative process [1, 2], in which lattice vibrations assist the motion of protons between neighboring oxygen sites. In acceptor‐doped perovskite oxides ${\mathrm{ABO}}_{3-\delta }$, oxygen vacancies $V_{O}^{••}$ are introduced for charge compensation and are filled upon exposure to water vapor via the hydration reaction

(1) $$H2O\left(g\right)+V_{O}^{••}+O_{O}^{\times }->2OH_{O}^{•},$$

incorporating protons as hydroxyl defects on lattice oxygen sites. Recently it was shown that excitation of OH vibrations in hydrated proton conductors could considerably increase the proton conductivity [3, 4]. While local OH/OD stretching and bending modes are well characterized by infrared and Raman spectroscopy, long‐range proton transport is governed by collective distortions of the ${\mathrm{BO}}_{6}$ framework that modulate transfer and reorientation barriers. Proton transport in perovskite oxides has been addressed both in early theoretical work emphasizing proton–lattice coupling and in later studies focusing on thermally activated transport in specific perovskite materials, notably by Kreuer and co‐workers [5]. Despite decades of intensive research, the identification of the true conducting protonic species in perovskite oxides remains challenging, in particular because surface hydroxyl species, physisorbed water, and bulk mobile protons can easily be confused by common spectroscopic techniques [6].

Nuclear resonance vibrational spectroscopy (NRVS) provides element‐specific access to the vibrational density of states by selectively probing the motion of a chosen atomic species [7]. In the case of ${\mathrm{BaSnO}}_{3}$‐based proton conductors, 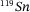
‐NRVS is sensitive to the Sn‐projected phonon spectrum and therefore does not directly probe proton dynamics. However, this selectivity enables a complementary perspective, as it provides direct insight into how proton incorporation perturbs the host lattice dynamics of the Sn–O framework. In this way, NRVS allows one to quantify the lattice response to proton incorporation, rather than the proton motion itself.

Our NRVS results provide direct experimental evidence for lattice‐assisted proton dynamics predicted for ${\mathrm{BaSnO}}_{3}$, including thermally assisted quantum motion in coincidence configurations [8]. Early theoretical work has emphasized that mobile protonic defects in hydrogen‐bonded systems behave as strongly coupled quasiparticles interacting with collective lattice vibrations [9].

Here, we apply 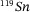
‐NRVS to nominally dry and hydrated ${\mathrm{BaSn}}_{0.8}Y_{0.2}O_{3-\delta }$ ($H_{2}O$ and $D_{2}O$) and analyze the resulting PDOS in terms of its first spectral moment and selected energy windows relevant to proton‐coupled lattice motion.

Recent results indicate that Y incorporation into the lattice leads to a pronounced softening of specific phonon modes, as identified by phonon calculations and supported experimentally by nuclear resonance vibrational spectroscopy (NRVS) and Raman spectroscopy, which probe the vibrational density of states rather than the full phonon dispersion [10, 11]. This softening is associated with a reduction of the activation energy for proton transport.

The system approaches a regime of reduced dynamical stability, reflected in lowered vibrational frequencies of the relevant lattice modes. The experimental basis for these findings was provided by nuclear resonance vibrational spectroscopy (NRVS) [7], which yields element‐specific phonon densities of states. Because NRVS requires the observed element to possess a Mössbauer transition, ${\mathrm{BaSnO}}_{3}$ was selected as a model proton‐conducting system with 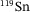 as the resonant isotope [12, 13].

Ionic conductivities of ceramic electrolytes are typically determined by electrochemical impedance spectroscopy under varying temperatures and gas atmospheres ($O_{2}$, $N_{2}$, Ar, $H_{2}$) [14]. Ion mobility can also be probed by quasi‐elastic neutron scattering (QENS), which provides access to proton diffusivity and its activation energy [15, 16, 17].

QENS studies have shown that at low temperatures, when protons remain bound to lattice oxygens, their mobility is characterized by very small activation energies (a few meV), consistent with local rotational motion around an oxygen site. At higher temperatures, protons can detach and hop to neighboring oxygen sites, resulting in long‐range proton conduction.

Molecular‐dynamics simulations [18, 19] have mapped the resulting proton trajectories and optimal diffusion paths in numerous perovskite systems. Additional work has proposed that lattice vibrations themselves propel proton motion, through coupled wagging or bending modes, such that the bound proton behaves as a “proton polaron” in the elastic field of the lattice [1, 20]. The vibrational properties of solids are commonly described by the phonon density of states (PDOS), accessible through various spectroscopy methods. Infrared and Raman techniques probe the Γ‐point of the Brillouin zone (BZ), whereas inelastic neutron scattering (INS) can sample the entire BZ in single crystals, yielding a full PDOS. However, these methods do not distinguish which atomic sublattices contribute to the observed modes and thus leave it open how particular sites and elements contribute to the PDOS. NRVS uniquely provides element‐specific PDOS information, limited to isotopes with Mössbauer transitions, but still highly valuable for benchmarking first‐principles lattice‐dynamical calculations such as DFT simulations [21, 22]. Here we focus on yttrium substituted barium stannate (BSY), extending our recent NRVS study on dry barium stannate [11] to hydrated and deuterated specimens. In the hydrated state, protons bind to lattice oxygens OH and become part of the stoichiometry; upon heating they detach and contribute to conductivity as mobile protonic defects (proton polarons). We show that the 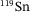
‐projected PDOS provides a sensitive probe of this lattice–proton coupling.

We place our findings in the broader context of hydrogen dynamics previously revealed by NRVS in biological systems, most notably in the study “How Nitrogenase Shakes” [23]. While the biochemical context differs fundamentally from solid‐state proton transport, both systems demonstrate that light hydrogen atoms couple to collective heavy‐atom vibrations, manifesting primarily as a redistribution of vibrational spectral weight rather than as isolated local modes.

The goals of this work are to: (i) quantify hydration‐induced changes in the Sn‐projected phonon density of states, (ii) assess whether H/D substitution produces a detectable isotope effect in the collective lattice dynamics, and (iii) relate these observations to proton–lattice coupling in proton‐conducting perovskite oxides.

## 2 Results and Discussion

Neutron diffraction and anomalous X‐ray diffraction confirm that ${\mathrm{BaSnO}}_{3}$ and Y‐substituted ${\mathrm{BaSn}}_{1-x}Y_{x}O_{3-\delta }$ (x=0.1,0.2) crystallize in the cubic perovskite structure (space group $Pm\bar{3}m$) without detectable symmetry lowering or long‐range ordering. Hydration and deuteration do not introduce additional Bragg reflections or peak splitting, indicating that OH/OD incorporation affects the structure only locally. ${\mathrm{BaSn}}_{0.9}Y_{0}.1O_{3-\delta }$ has some slight lattice expansion (4.150 Å dry, 4.153 Å $H_{2}O$ and $D_{2}O$). For ${\mathrm{BaSn}}_{0.8}Y_{0.2}O_{3-\delta }$, the lattice parameter determined by laboratory X‐ray diffraction is a=4.162 Å.

Anomalous X‐ray diffraction across the Sn and Y absorption edges shows that both cations occupy the same crystallographic B‐site and that no Sn‐ or Y‐rich secondary phases are present. Weak diffuse intensity under Y‐resonant conditions points to short‐range Y‐induced local distortions, but no commensurate superstructures are observed. The perovskite framework thus remains intact, and hydration‐induced effects must be accommodated primarily by local distortions and force‐constant variations rather than by changes of crystal symmetry.

**FIGURE 1:**
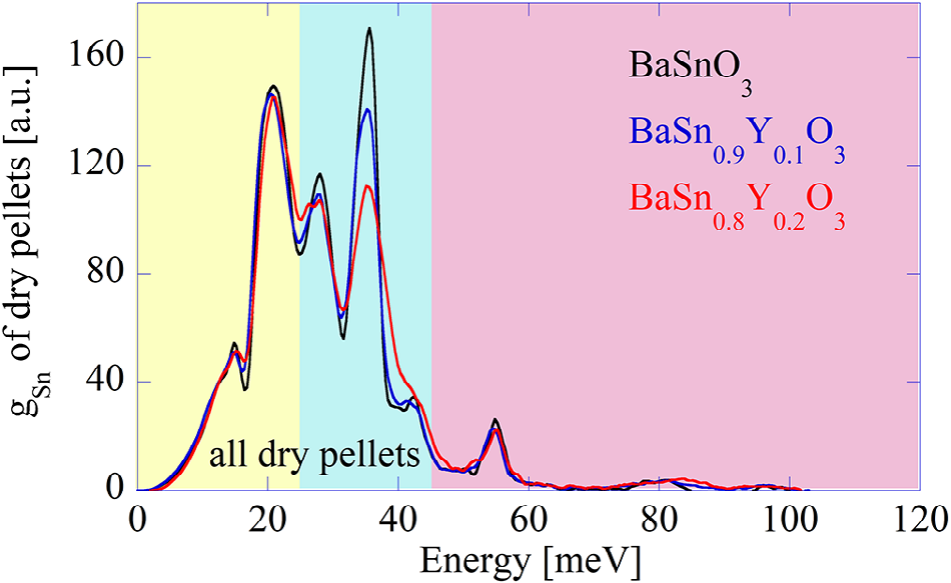
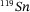
‐projected PDOS of dry ${\mathrm{BaSnO}}_{3}$ and Y‐doped variants. Comparison of ${\mathrm{BaSnO}}_{3}$, ${\mathrm{BaSn}}_{0.9}Y_{0.1}O_{3}$, and ${\mathrm{BaSn}}_{0.8}Y_{0.2}O_{3}$ in the nominally dry state at 300 K. The colored regions roughly indicate acoustic/tilt modes (yellow), Sn–O bending and mixed modes (blue), and higher‐energy stretching‐dominated contributions (pink).

**FIGURE 2:**
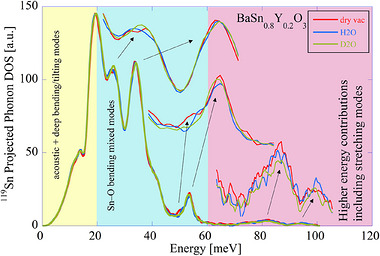
Isotopic mass effects in hydrated BSY20. 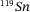
‐projected PDOS of ${\mathrm{BaSn}}_{0.8}Y_{0.2}O_{3-\delta }$ (BSY20) in the nominally dry, $H_{2}O$‐hydrated, and $D_{2}O$‐hydrated states at 300 K. The colored regions indicate acoustic/tilt modes (yellow), Sn–O bending and mixed modes (blue), and higher‐energy stretching‐dominated contributions (pink).

**FIGURE 3:**
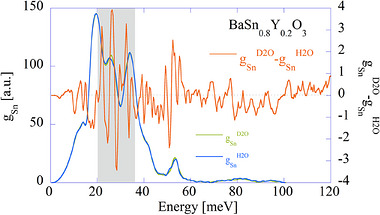
Isotope‐induced reweighting of the PDOS in BSY20. Difference spectrum $g_{\mathrm{Sn}}^{D}2O-g_{\mathrm{Sn}}^{H}2O$ for ${\mathrm{BaSn}}_{0.8}Y_{0.2}O_{3-\delta }$ (BSY20), highlighting isotope‐sensitive spectral reweighting. The shaded band marks the 20 to 35 meV interval, where the changes are most pronounced.

**FIGURE 4:**
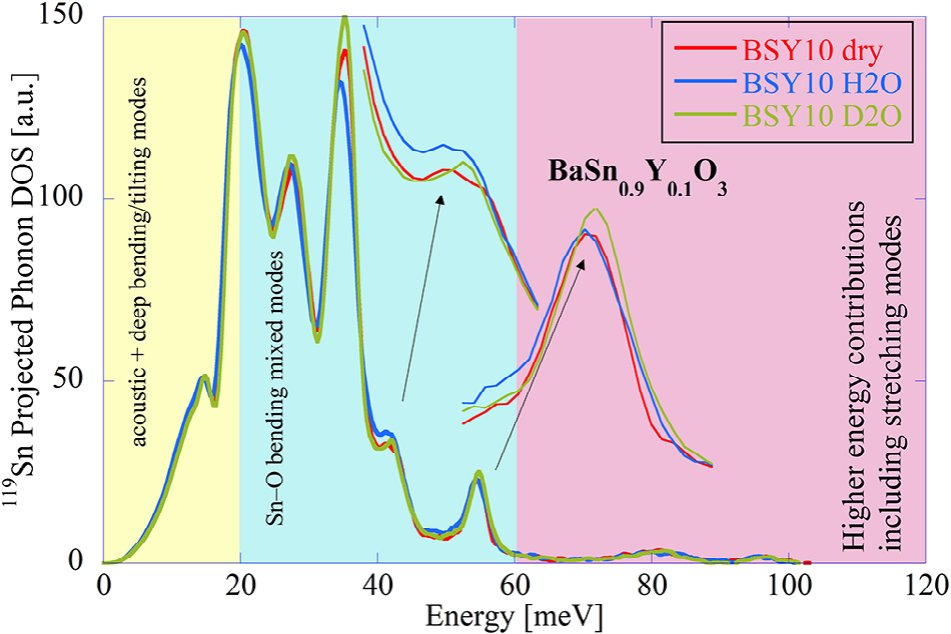
Hydration and isotope effects on the PDOS of BSY10. 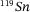
‐projected PDOS of ${\mathrm{BaSn}}_{0.9}Y_{0.1}O_{3-\delta }$ (BSY10) in the nominally dry, $H_{2}O$‐hydrated, and $D_{2}O$‐hydrated states at 300 K. The colored regions indicate acoustic/tilt modes (yellow), Sn–O bending and mixed modes (blue), and higher‐energy stretching‐dominated contributions (pink).

**FIGURE 5:**
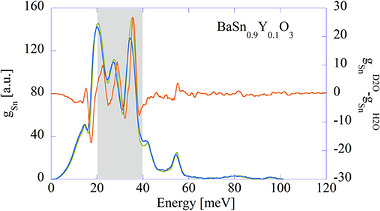
Isotope‐induced reweighting of the PDOS in BSY10. Difference spectrum $g_{\mathrm{Sn}}^{D}2O-g_{\mathrm{Sn}}^{H}2O$ for ${\mathrm{BaSn}}_{0.9}Y_{0.1}O_{3-\delta }$ (BSY10), highlighting isotope‐sensitive spectral reweighting. The shaded band marks the 20 to 35 meV interval, where the changes are most pronounced.

The NRVS dataset is organized as follows. Figure 1 establishes the dry‐state baseline by comparing ${\mathrm{BaSnO}}_{3}$, ${\mathrm{BaSn}}_{0.9}Y_{0.1}O_{3}$ and ${\mathrm{BaSn}}_{0.8}Y_{0.2}O_{3}$ and defines three characteristic energy windows. Figures 2 and 3 then document hydration‐ and isotope‐dependent changes in the more defect‐rich composition ${\mathrm{BaSn}}_{0.8}Y_{0.2}O_{3-\delta }$ (BSY20). Figures 4 and 5 provide the corresponding dataset for the lower‐doped composition ${\mathrm{BaSn}}_{0.9}Y_{0.1}O_{3-\delta }$ (BSY10). This sequence separates (i) baseline doping effects in the dry lattice from (ii) hydration‐ and isotope‐ induced spectral reweighting, enabling a direct comparison of defect‐controlled broadening and isotope sensitivity between BSY10 and BSY20. The composition ${\mathrm{BaSn}}_{0.8}Y_{0.2}O_{3-\delta }$ was selected to provide a high concentration of oxygen vacancies, enabling substantial proton incorporation upon hydration. An yttrium substitution level of 20% represents a well‐established compromise between maximizing proton uptake and preserving the structural stability of the perovskite lattice, thereby allowing measurable changes in lattice dynamics.

The corresponding NRVS raw spectra recorded for dry ${\mathrm{BaSnO}}_{3}$ as well as during in situ hydration with $H_{2}O$ and $D_{2}O$ are provided in Figures S5–S7. Normalized isotope‐difference spectra for BSY20 and BSY10 are shown in Figures S8 and S9, respectively. Figure  S10 presents a heatmap representation of the Sn‐projected PDOS, summarizing the evolution from dry to $H_{2}O$‐ and $D_{2}O$‐hydrated ${\mathrm{BaSnO}}_{3}$. The visualization highlights systematic changes in spectral weight upon hydration as well as clear isotope‐dependent shifts, providing direct evidence for proton‐mass–dependent coupling to lattice vibrations. The first spectral moment was evaluated for each individual scan underlying the averaged spectra, yielding a standard error of 0.04–0.05 meV, confirming that the observed hydration‐induced shift ( 0.3–0.4 meV) is well resolved.

Figure 1 compares the 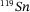
‐projected PDOS of nominally dry pellets of ${\mathrm{BaSnO}}_{3}$, ${\mathrm{BaSn}}_{0.9}Y_{0.1}O_{3}$, and ${\mathrm{BaSn}}_{0.8}Y_{0.2}O_{3}$ at 300 K. Three spectral regions are highlighted: (i) acoustic and low‐energy tilt/bending modes below ∼20 meV, (ii) mixed Sn–O bending and collective modes in the 20–60 meV range, and (iii) higher‐energy stretching‐related contributions above ∼60 meV.

Across all dry samples (${\mathrm{BaSnO}}_{3}$, ${\mathrm{BaSn}}_{0.9}Y_{0.1}O_{3}$, ${\mathrm{BaSn}}_{0.8}Y_{0.2}O_{3}$) and across all measurement campaigns (2022–2024), the low‐energy region from 15–25 meV with acoustic and collective lattice modes are essentially identical. This behavior reflects the dominance of the Ba–O framework in this range and confirms that a 10%–20% B‐site substitution on Sn has only a minor effect on the collective low‐frequency dynamics. In the mid‐energy range of 25–45 meV, the spectra show subtle but systematic, chemically meaningful differences. The mixed Sn–O bending and stretching modes exhibit a slight hardening with increasing Y content (typically +0.3 to +0.8 meV) together with changes in the relative peak intensities. These trends arise from the reduced effective mass ($Y^{3+}$ vs. ${\mathrm{Sn}}^{4+}$), local distortions of the ${\mathrm{BO}}_{6}$ octahedra, and the onset of partial mode localization around Y‐rich environments. In the high energy region of 50 to 65 meV, the Y‐doped samples—most notably ${\mathrm{BaSn}}_{0.8}Y_{0.2}O_{3}$—consistently display increased spectral weight in the high‐energy O‐dominated part of the PDOS (around 55 to 60 meV). This additional shoulder is physically plausible and reflects the perturbation of the local Sn–O force‐constant landscape by Y substitution, leading to partial mode splitting and a broader distribution of high‐frequency states. Results of the quantitative statistical analyzes of the PDOS are listed in Table 1.

**TABLE 1:** Energy moments of the PDOS for the dry pellets (E≥0 meV).

Sample	⟨E⟩ (meV)	$\left\langle E^{2}\right\rangle$ (${\mathrm{meV}}^{2}$)	Var(E) (${\mathrm{meV}}^{2}$)	σ (meV)
${\mathrm{BaSnO}}_{3}$	27.46	793.0	39.3	6.27
${\mathrm{BaSn}}_{0.9}Y_{0.1}O_{3}$	28.06	893.0	105.6	10.28
${\mathrm{BaSn}}_{0.8}Y_{0.2}O_{3}$	28.23	905.6	108.7	10.43

The increase in the second statistical moment and variance of the PDOS upon Y substitution in ${\mathrm{BaSn}}_{1-x}Y_{x}O_{3}$ provides direct evidence for a broader distribution of local vibrational environments. While the mean phonon energy ⟨E⟩ increases only slightly (from 27.46 to 28.23 meV), the variance grows by nearly a factor of three, indicating a substantial broadening of the phonon spectrum. Three physical factors contribute to this behavior. First, heterovalent substitution (${\mathrm{Sn}}^{4+}\to$ $Y^{3+}$) introduces local structural disorder. Differences in ionic radius, charge, and bonding character distort the ${\mathrm{BO}}_{6}$ octahedra and generate a distribution of Sn–O and Y–O bond lengths and corresponding force constants. This disorder naturally broadens the phonon energies. Second, with increasing Y content, a fraction of vibrational modes becomes partially localized around Y‐rich environments. The resulting symmetry breaking splits previously degenerate Sn–O bending and stretching vibrations, producing additional states on both the low‐ and high‐energy sides of the spectrum and thereby increasing Var(E). Third, the high‐energy O‐dominated modes (50–65 meV) acquire additional spectral weight in the doped samples, especially for x=0.2. Because the second moment $\left\langle E^{2}\right\rangle$ is weighted by $E^{2}$, even moderate intensity in this high‐energy tail significantly enhances the variance. Taken together, the increasing variance of the PDOS serves as a quantitative fingerprint of the growing structural and dynamical disorder in ${\mathrm{BaSn}}_{1-x}Y_{x}O_{3}$, reflecting local force‐constant heterogeneity and partial mode localization induced by Y substitution.

Figure 2 shows the 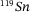
‐projected PDOS of ${\mathrm{BaSn}}_{0.8}Y_{0.2}O_{3-\delta }$ (BSY20) in the nominally dry (“dry vac”), $H_{2}O$‐hydrated, and $D_{2}O$‐hydrated states at 300 K. The overall band positions remain similar across the three states, while reproducible differences appear as changes in intensity and fine structure, most notably in the 20–35 meV window and above ∼60 meV.

The key vibrational parameters extracted from the NRVS spectra are summarized in Table 2. In particular, the low‐energy phonon modes exhibit a systematic softening upon hydration, accompanied by a mass‐dependent shift consistent with proton incorporation. These quantitative trends form the basis for the discussion of proton–phonon coupling below.

**TABLE 2:** Representative peak positions in the Sn‐projected PDOS of ${\mathrm{BaSn}}_{0.8}Y_{0.2}O_{3-\delta }$ (BSY20) for $H_{2}O$‐ and $D_{2}O$‐hydrated samples.

$H_{2}O$			$D_{2}O$		
Energy (meV)	DOS	Prominence	Energy (meV)	DOS	Prominence
19.8	145.0	32.0	19.6	140.1	140.1
25.9	132.0	28.0	26.0	105.4	8.99
34.7	118.0	24.0	34.4	107.3	53.85
53.5	82.0	18.0	53.2	22.6	14.43

To visualise the isotope‐sensitive redistribution more directly, Figure 3 shows the difference spectrum $\left(g_{\mathrm{Sn}}^{D}2O-g_{\mathrm{Sn}}^{H}2O\right)$ for BSY20. The dominant response is concentrated in the 20 to 35 meV range, indicating that H/D substitution primarily reweights spectral intensity among closely spaced Sn–O bending and mixed lattice modes rather than generating new features in the Sn‐projected spectrum.

Figure 4 provides the corresponding dataset for ${\mathrm{BaSn}}_{0.9}Y_{0.1}O_{3-\delta }$ (BSY10). Compared to BSY20, the Sn–O‐related bands in the 25–45 meV range are noticeably sharper and better resolved, consistent with reduced inhomogeneous broadening at lower defect concentration.

Figure 5 shows the isotope difference spectrum for BSY10. The difference signal is highly structured and is primarily confined to the 20 to 35 meV range, i.e. to Sn–O bending and mixed collective modes. The rapid decay of the difference above around 50 meV indicates that the isotope response remains dominated by low‐ and mid‐energy lattice modes.

Across both compositions, hydration and H/D substitution primarily redistribute spectral weight among existing Sn‐coupled lattice modes, with the clearest isotope sensitivity in the 20–35 meV band. The isotope difference spectra are resolved more cleanly in BSY10, while in BSY20 the same response is partially masked by stronger inhomogeneous broadening associated with higher defect and proton concentrations.

To further assess relative spectral changes across the full energy range, we also evaluated normalized isotope‐difference spectra, $\left(g^{D}2O_{\mathrm{Sn}}-g^{H}2O_{\mathrm{Sn}}\right)/g_{\mathrm{Sn}}^{\mathrm{dry}}$, which are shown in the Figures S8 and S9. While the absolute difference spectra emphasize the dominant isotope response in the 20–35 meV range, they can underrepresent relative variations in spectral regions where the dry Sn‐projected PDOS is small. The normalized representation therefore provides a complementary view of relative changes across the full spectral range. At the same time, in regions where $g_{\mathrm{Sn}}^{\mathrm{dry}}$ approaches zero, the normalization becomes noise‐amplified and does not provide a robust basis for quantitative interpretation. Taken together, the absolute and normalized spectra show that the dominant isotope‐dependent response remains confined to the low‐ and mid‐energy Sn–O lattice modes, whereas higher‐energy variations remain minor in absolute magnitude and do not alter the overall physical interpretation.

### 2.1 Structural Integrity and Local Distortions

Neutron and anomalous X‐ray diffraction, as shown in the Figures S1–S3, establish that ${\mathrm{BaSnO}}_{3}$ and Y‐substituted ${\mathrm{BaSn}}_{1-x}Y_{x}O_{3-\delta }$ retain the cubic perovskite structure upon hydration and deuteration, without detectable symmetry lowering or long‐range ordering. The absence of additional Bragg reflections or peak splitting indicates that proton incorporation and isotopic substitution do not induce structural phase transitions on the length scales accessible to diffraction. Instead, the structural response to hydration is confined to the local scale, involving subtle distortions and variations of local bonding environments around oxygen vacancies and hydroxylated sites.

These observations provide an essential framework for interpreting the NRVS results: the isotope‐dependent changes observed in the PDOS cannot be attributed to crystallographic transformations, but must originate from local modifications of force constants and lattice dynamics within an otherwise intact perovskite framework.

Compared to ${\mathrm{BaSn}}_{0.8}Y_{0.2}O_{3-\delta }$, the 10% Y‐doped sample ${\mathrm{BaSn}}_{0.9}Y_{0.1}O_{3-\delta }$ exhibits markedly sharper and more clearly resolved Sn–O phonon bands, most prominently at 27, 34, and 42 meV (Figure 4). This reflects a narrower distribution of local Sn–O force constants and a more homogeneous lattice environment at lower defect concentration. The corresponding isotope difference spectrum $g_{\mathrm{Sn}}^{D}2O-g_{\mathrm{Sn}}^{H}2O$ (Figure 5) is highly structured and largely confined to the 20–35 meV range, where collective Sn–O bending and mixed lattice modes dominate. The oscillatory sign changes indicate a redistribution of spectral weight among closely spaced phonon branches rather than a uniform frequency shift, while the rapid decay of the difference above ∼50 meV shows that the isotope sensitivity is restricted to low‐ and mid‐energy lattice modes. Compared to BSY20, the difference signal in BSY10 is both stronger and more clearly resolved, indicating a more coherent dynamic coupling between protonic degrees of freedom and the Sn–O framework at lower defect concentration.

In this sense, the hydrated and deuterated lattices provide an experimental realization of the dynamic coupling between light interstitial species and the heavy‐atom framework that is anticipated by theory. The trends identified here form the experimental basis for the oscillator–crystal–defect description developed below.

In this respect, the present results provide a crystalline solid‐state counterpart to earlier NRVS studies on biological metalloenzymes [23], where hydrogen motion was likewise shown to modulate collective metal‐centered vibrations rather than forming independent local oscillators.

The 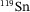
‐projected PDOS reveals that hydration and H/D substitution primarily affect the distribution of spectral weight among existing lattice modes rather than generating new vibrational features. Because NRVS selectively probes Sn‐weighted vibrations, internal OH/OD stretching modes contribute only weakly to the measured spectra. Instead, the observed isotope sensitivity reflects how light interstitial species perturb collective Sn–O bending and mixed lattice modes.

The modest isotope‐dependent frequency shifts and intensity redistributions, which deviate systematically from the ideal $1/\sqrt{2}$ mass scaling, demonstrate that proton incorporation modifies the local stiffness landscape of the lattice in addition to changing effective masses. Hydrogen therefore acts as a dynamically coupled perturbation of the Sn–O framework rather than as an independent local oscillator. This behavior is consistent with the cooperative nature of proton conduction in oxide perovskites, where long‐range transport is governed by collective lattice distortions that mediate proton transfer and reorientation [24].

The modest isotope dependence observed in the Sn‐projected PDOS is consistent with a picture in which proton dynamics are governed by coupling to collective lattice modes, rather than by localized protonic vibrations. Such correlated proton–phonon dynamics have been directly observed in other solid‐state systems [25].

Importantly, unlike the biological case [23], the present system allows for controlled defect chemistry, isotopic substitution, and quantitative moment analysis of the PDOS, enabling a direct experimental assessment of proton–lattice coupling in a periodic solid.

### 2.2 Defect‐Controlled Lattice Inhomogeneity: BSY10 vs. BSY20

A comparison of ${\mathrm{BaSn}}_{0.9}Y_{0.1}O_{3-\delta }$ and ${\mathrm{BaSn}}_{0.8}Y_{0.2}O_{3-\delta }$ highlights the role of defect concentration in shaping lattice dynamics. At lower Y content (BSY10), the Sn‐projected phonon bands remain relatively sharp and well defined, indicating a more homogeneous distribution of local force constants. In contrast, the higher Y concentration in BSY20 leads to pronounced broadening of the same modes, reflecting increased chemical and dynamical inhomogeneity associated with higher defect and proton concentrations.

Despite these differences, the underlying lattice dynamics remain of the same character across both compositions: hydration and isotopic substitution reweight collective Sn–O modes without introducing new vibrational degrees of freedom. Increasing Y content thus amplifies the extent of lattice inhomogeneity rather than altering the fundamental mechanism of proton–lattice coupling. Together, these results identify isotope‐sensitive reweighting of collective phonons as a robust experimental signature of defect‐mediated proton–lattice interactions in proton‐conducting perovskite oxides.

### 2.3 Hydration‐Induced Stiffening of the Sn–O Framework

The PDOS analysis shows that hydration leads to a collapse of the double‐peak structure in the 22–27 meV region into a broader, more symmetric band and to a small but systematic increase of the first spectral moment $M_{1}$. Both effects indicate a net stiffening of the Sn–O vibrational potential when oxygen vacancies are filled with hydroxyl groups. Microscopically, this can be understood in terms of a more regular octahedral connectivity and reduced configurational disorder in the hydrated lattice.

The trend is consistent with our earlier Raman experiments on dry and hydrated ${\mathrm{BaZrYO}}_{3}$,[26] where compressive strain and hydration shifted characteristic lattice modes to higher frequencies. In the present perovskite stannates, formation of O–H bonds locally modifies the Coulomb interactions and reduces the polarizability of the oxygen sublattice, thereby hardening the relevant Sn–O phonon modes. The isotope analysis, expressed via r and Δω/ω, then quantifies these stiffness changes in terms of Δk/k.

Hydration fills oxygen vacancies and forms hydroxyl groups, introducing locally strong and directional O–H bonds. While these O–H vibrations lie outside the spectral window of the Sn‐projected PDOS, their presence modifies the local force‐constant landscape of the lattice. Spectra of dry and hydrated BCY10 obtained from inelastic neutron scattering (INS) at 723 K (Figure S11) show a broad contribution around ∼27 meV that is strongly reduced upon hydration, consistent with the removal of oxygen‐vacancy‐related lattice modes. A similar hydration‐induced spectral evolution has been observed in yttrium‐ substituted barium cerate using operando (near‐) ambient pressure XPS (compare [27]).

Complementary INS measurements confirm that hydration modifies the lattice dynamics in the same energy range (20–35 meV) where the Sn‐projected PDOS shows isotope‐sensitive reweighting. In addition, a pronounced quasielastic contribution at low energies indicates the presence of mobile protonic species.

The removal of oxygen vacancies eliminates locally under‐coordinated, mechanically softer regions, while hydroxyl formation stabilizes the oxygen sublattice through directional bonding. As a result, the lattice becomes more homogeneous and effectively stiffer, which is reflected in the observed increase of the first spectral moment and the redistribution of spectral weight in the 20–35 meV range.

Thus, the hydration‐induced stiffening observed in the Sn‐weighted PDOS does not arise from direct contributions of O–H vibrational modes, but from their indirect influence on the collective lattice dynamics.

### 2.4 Why H/D Substitution Barely Shifts the Sn‐PDOS

The near‐identical first moments $M_{1}$ of the $H_{2}O$‐ and $D_{2}O$‐hydrated samples may at first seem surprising given the clear mass difference between H and D. However, this behavior is consistent with the nature of the NRVS observable. 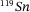
‐NRVS probes the kinetic energy of vibrations weighted by the partial mean‐square displacements of Sn. Collective Sn–O modes involve an effective mass dominated by Sn and O; replacing H by D in local OH/OD groups changes the protonic mass but only weakly perturbs the Sn‐centred collective coordinates. Consequently, H/D substitution primarily affects local OH/OD modes (readily seen in optical Raman spectra as a $∼1/\sqrt{2}$ shift of the bending and stretching frequencies), while the Sn‐weighted collective modes remain nearly unchanged in energy and only redistribute spectral weight at the percent level. In this sense, NRVS and Raman spectroscopy provide complementary perspectives: NRVS constrains the collective Sn–O part of the Hamiltonian, whereas Raman on OH/OD modes constrains the local protonic part. Together they realise a minimal empirical example of a proton–phonon vibronic oscillator in a ceramic proton conductor.

### 2.5 Structural and Electronic Context: Resonant Diffraction and XPS/UPS

Resonant powder diffraction, as shown in the Figures S2 and S3, confirms that ${\mathrm{BaSnO}}_{3}$ and ${\mathrm{BaSn}}_{0.9}Y_{0.1}O_{3}$ retain essentially single‐phase perovskite structures and show clear element‐specific scattering at the Sn and Y K edges. ${\mathrm{BaSn}}_{0.9}Y_{0.1}O_{3}$ exhibits a markedly stronger energy dependence than ${\mathrm{BaSnO}}_{3}$, reflecting Y‐driven modifications of the local Sn–O coordination and the emergence of short‐range Sn–O–Y distortions. No intense superstructure reflections are detected, but weak, energy‐dependent shoulders and minor intensity redistributions in the Y‐containing material are consistent with short‐range structural motifs associated with oxygen‐vacancy complexes and their hydration. These structural observations dovetail with the electronic and vibrational probes. XPS (Figure S4) resolves chemically distinct oxygen environments, UPS reveals corresponding defect states near the valence band, and the Sn‐projected PDOS captures their vibrational fingerprints.

Taken together, the data support a picture in which Y doping creates structurally and electronically heterogeneous sites that serve as nucleation centres for hydration and stabilize O–H species. These local motifs define the structural origin of the protonic defects, while the resulting changes in the phonon landscape give rise to the collective low‐energy vibrational modes that underpin the hydration‐induced phonon renormalization.

Consistent with our recent findings [10], the Y‐induced distortions act as the structural seed for the imaginary‐mode behavior that lowers the activation barrier for proton migration. Earlier resonant X‐ray and neutron diffraction results on Y‐substituted barium zirconate [28] have shown that Y atoms are not randomly distributed but arrange into superstructural motifs, which in turn imprint preferential pathways for proton motion across the lattice–analogous to predefined passes laid into a mountainous landscape. Hydration therefore does not generate a uniform protonic medium but activates a pre‐patterned network of energetically favorable sites and links.

Such structurally conditioned landscape also provides the natural setting for polaronic charge transport. As shown previously for hydrated barium zirconate, the proton interacts strongly with the surrounding lattice, forming a local protonic polaron whose dynamics are governed by coupling to specific phonon modes [1]. The integration of these observations suggests that proton transport in ${\mathrm{BaSn}}_{1-x}Y_{x}$ is dynamically enabled but structurally originated: local Y‐induced environments create the conditions, the superstructure prescribes the pathways, and the phonon field provides the mechanism.

## 3 Conclusion

We have investigated the effect of hydration and isotopic substitution on the 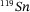
‐projected PDOS of ${\mathrm{BaSn}}_{0.8}Y_{0.2}O_{3-\delta }$ by NRVS at 300 K. A statistical analysis of the first spectral moment $M_{1}$ and selected energy windows shows that hydration leads to a measurable stiffening of the Sn–O framework, whereas the difference between $H_{2}O$‐ and $D_{2}O$‐hydrated samples remains within the experimental uncertainty. Normalization of the vibrational difference spectra confirms that proton‐induced modifications are primarily confined to the low‐energy lattice modes, with only minor relative variations at higher energies that do not alter the overall physical interpretation. This is fully consistent with the collective, Sn‐weighted nature of the NRVS observable and with a simple proton–phonon oscillator model in which hydration modifies the Sn–O force constants while H/D substitution primarily affects local OH/OD vibrations. Taken together, the present results suggest that a combined analysis of element‐specific PDOS and proton‐sensitive spectroscopies can provide quantitative insight into the vibronic mechanisms underlying proton transport in perovskite oxides. Viewed more broadly, these results extend the concept of hydrogen‐driven collective vibrations–previously observed in complex biological systems– to a well‐defined crystalline solid, where defect chemistry and lattice dynamics can be tuned and quantified independently.

Across both BSY10 and BSY20, hydration and H/D substitution do not generate new Sn‐projected vibrational modes, but instead redistribute spectral weight within existing collective Sn–O lattice modes, most clearly in the 20–35 meV range. The lower‐doped composition BSY10 exhibits sharper and more clearly resolved isotope‐dependent features, indicating a more homogeneous local force‐constant landscape, whereas BSY20 shows stronger inhomogeneous broadening associated with its higher defect and proton concentration. Despite these differences in spectral resolution, both compositions support the same physical picture: proton incorporation perturbs the collective lattice dynamics of the Sn–O framework, rather than behaving as an isolated local oscillator within the low‐energy lattice‐dynamical regime probed here. We note that localized O–H stretching modes at higher energies are well established; however, recent work demonstrates that these modes are dynamically coupled to the lattice and can relax into collective vibrations that modulate proton transport [4].

Within a coupled proton–phonon oscillator model, the observed spectral changes can be rationalized in terms of a renormalization of effective force constants and vibrational masses of the Sn–O framework upon proton incorporation. The model quantitatively reproduces (i) the hydration‐induced increase of the first spectral moment, (ii) the isotope‐dependent redistribution of spectral weight without the appearance of new vibrational modes, and (iii) the sensitivity of the Sn‐projected PDOS to proton‐induced lattice perturbations. Together, these results support a picture of proton transport governed by coupled, collective lattice dynamics rather than localized vibrational modes.

## 4 Experimental Section

### 4.1 Materials Synthesis and Hydration Protocol

Polycrystalline ${\mathrm{BaSn}}_{0.8}Y_{0.2}O_{3-\delta }$ was synthesized by a method involving metal 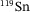 as precursor, as detailed in the Supporting Information. Using stoichiometric amounts of high‐purity ${\mathrm{BaCO}}_{3}$, ${\mathrm{SnO}}_{2}$ and $Y_{2}O_{3}$, and calcination at 1673 K and intermediate grindings, the powders were pressed into pellets and sintered at 1973 K. The oxygen deficiency δ and the Sn valence state were verified by XPS and neutron diffraction. Dry samples were obtained by storing the pellets at elevated temperature 800 K under dry gas flow or dynamic vacuum, followed by cooling under dry conditions. Hydrated samples were prepared by exposing dry pellets to flowing $H_{2}O$ or $D_{2}O$ vapour at 350 K for sufficient time to reach equilibrium. The hydration state was monitored by mass uptake. For the present NRVS study we focus on the following states: nominally dry, $H_{2}O$‐hydrated, and $D_{2}O$‐hydrated ${\mathrm{BaSn}}_{0.8}Y_{0.2}O_{3-\delta }$.

### 4.2 Structure Analysis by Neutron Diffraction

Neutron powder diffractograms were recorded [11] at the HRPT neutron beamline at the Swiss Spallation Neutron Source in Villigen, Switzerland [29, 30, 31]. For neutron diffraction, we used powder samples, prepared as described above from natural‐abundance isotope tin foil from Goodfellow. Diffractograms were acquired at a neutron wavelength of 1.1545 Åat temperatures of 1, 100, and 200 K. Structure refinement was performed with the GSAS‐II package [32]. Details of the neutron diffraction experiment and full refinements are given in [10]. High–resolution neutron powder diffraction (ND) was used to establish the crystallographic baseline and vibrational displacement parameters of the ${\mathrm{BaSn}}_{0.8}Y_{0.2}O_{3-\delta }$ (BSY20) samples. The full refinements, experimental details, and temperature–dependent structural parameters are reported in a companion manuscript in [10]. Here we summarize the aspects directly relevant to the vibrational analysis and to the interpretation of the isotope–dependent NRVS results.

### 4.3 Structure Analysis by X‐Ray Diffraction

Resonant (anomalous) X‐ray powder diffraction measurements were carried out at beamline 12.2.2 at the Advanced Lightsource, Lawrence Berkeley National Laboratory, on ${\mathrm{BaSnO}}_{3}$ and ${\mathrm{BaSn}}_{0.9}Y_{0.1}O_{3}$. The experiment was designed as a feasibility test to (i) establish a robust calibration and integration workflow for hard X‐ray powder patterns of our samples and (ii) search for first indications of cation ordering or superstructure effects around the Y and Sn K absorption edges. Anomalous X‐ray diffraction experiments were carried out at photon energies tuned around the Sn K‐edge (28800, 29000, 29200, 29800 eV) and Y K‐edge (16000, 16900, 17000, 18000 eV) in order to selectively enhance the scattering contrast of the respective B‐site cations in ${\mathrm{BaSnO}}_{3}$, ${\mathrm{BaSn}}_{0.9}Y_{0.1}O_{3}$ (BSY10), and ${\mathrm{BaSn}}_{0.8}Y_{0.2}O_{3}$ (BSY20).

### 4.4 Ex Situ and In Situ NRVS


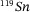
 nuclear resonance vibrational spectroscopy was performed at beamline BL35LXU at Spring‐8 in Hyogo, Japan [33, 34] using monochromatized synchrotron radiation tuned to the 23.875 keV nuclear resonance of 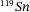. A series of 32 consecutive scans were recorded for each sample condition at 300 K. The energy step size and total scan range were 0.25 meV steps over ‐80 to +80 meV, with an instrumental energy resolution of approximately 1 meV (FWHM). The resolution was derived by fitting the elastic line with Gaussian peak; with σ = 0.64 (FWHM = 1.5, which agrees with the beamline resolution. For SBY20: First, the sample was measured in wet state under flow of wet nitrogen (90% relative humidity at 298 K) mixed with Humistat; then in vacuum, then the same with $D_{2}O$ (90% relative humidity at 298 K).

The raw NRVS spectra were processed using the NRVS.tool pipeline (spectra.tools) and PHOENIX‐3.0.6 software [35, 36] to obtain the 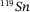
‐projected phonon density of states (PDOS). Fitting parameters were following: Temperature = 300K, fit range 2 meV, recoil energy 2.57 meV.

### 4.5 XPS and UPS Analysis of ${\mathrm{BaSnO}}_{3}$ and ${\mathrm{BaSn}}_{0.9}Y_{0.1}O_{3-x}$

High‐resolution X‐ray photoelectron spectroscopy (XPS) and ultraviolet photoelectron spectroscopy (UPS) were performed on ${\mathrm{BaSnO}}_{3}$ (BSO) and Y‐doped ${\mathrm{BaSn}}_{0.9}Y_{0.1}O_{3-x}$ (BSY10) to assess the chemical environments associated with Y substitution and hydration.

XPS measurements were carried out with charge neutralization using a low‐energy electron flood gun. Binding energies were referenced to adventitious carbon (C 1s = 284.8 eV). Owing to differences in surface morphology and charging behavior, small variations in peak positions and widths are expected.

For each material, three sample conditions were examined: (i) the as‐sintered top surface, (ii) a freshly fractured inner surface, and (iii) powder dispersed on carbon tape. In the present work, XPS/UPS data are used in a strictly qualitative manner to identify chemically distinct Sn, Y, and O environments and to support the interpretation of the vibrational spectroscopy results.

Complete XPS and UPS datasets, including detailed peak deconvolutions, quantitative analyzes, and surface‐condition comparisons, are provided in the data archive https://zenodo.org/records/18283055 and Supporting Information.

### 4.6 Generative AI Disclosure

Generative AI tools were used for language refinement and editorial clarity during manuscript preparation. All scientific content, data analysis, interpretation, and conclusions were developed, verified, and approved by the authors.

## Conflicts of Interest

The authors declare no conflicts of interest.

## Supporting information


**Supporting File**: advs76065‐sup‐0001‐SuppMat.pdf.

## Data Availability

The data that support the findings of this study are available from the corresponding author upon reasonable request.
