# Supplementary material for: How Proton Incorporation Reshapes Lattice Dynamics In BaSnO3‐Type Proton Conductors
Source: Adv Sci (Weinh). 2026 Jun 15;13(50):e76065. doi: 10.1002/advs.76065 (PMC13336920; doi:10.1002/advs.76065)
Supplement: Supplementary file 1 [file ADVS-13-e76065-s001.pdf]

How Proton Incorporation Reshapes Lattice Dynamics in $BaSnO_3$-type Proton Conductors

Artur Braun*[1], Alexey Rulev[1], Nobumoto Nagasawa[2], Hongxin Wang[3], Tatyana Bendikov[4], Vladimir Pomjakushin[5], Martin Kunz[6], Yoshitaka Yoda[2], Qianli Chen[7], and Stephen P. Cramer[3]

[1]Laboratory for High Performance Ceramics, Empa, Swiss Federal Institutes of Technology, CH - 8600 Dübendorf, Switzerland
[2]Precision Spectroscopy Division, SPring-8/JASRI, Sayo 679-5198, Hyogo, Japan
[3]SETI Institute, Mountain View, CA 94043, United States
[4]Department of Chemical Research Support, Weizmann Institute of Science, Rehovot, Israel
[5]Laboratory for Neutron Scattering, Paul Scherrer Institut, CH - 5232 Villigen PSI, Switzerland
[6]Advanced Lightsource, Lawrence Berkeley National Laboratory, Berkeley CA 94720, United States
[7]Global College, Shanghai Jiao Tong University, Shanghai, China

*Email: artur.braun@alumni.ethz.ch

Supporting Information

Materials synthesis (isotope-enriched tin)

Barium stannate ($BaSnO_3$) was prepared using a conventional ceramic solid–state route. For nuclear resonance vibrational spectroscopy (NRVS), the presence of a Mössbauer–active isotope is required; therefore, isotopically enriched ^{119}Sn was used as tin precursor. The enriched isotope is commercially available predominantly in metallic form. ^{119}Sn metal sheets were obtained from Neonest AB (BuyIsotope.com, Solna, Sweden), with a nominal enrichment of 96.3%.

For reference and comparison, tin metal with natural isotopic abundance (GoodFellow, 99.999% purity, CAS 7440–31–5), containing 8.59% ^{119}Sn, was employed to evaluate the effective isotope enrichment. The actual ^{119}Sn content of the enriched metal was independently verified by nuclear magnetic resonance (NMR), yielding a value of 84%.

For $BaSnO_3$ synthesis, the tin metal was dissolved in concentrated high–purity nitric acid (Sigma–Aldrich, 70%, redistilled, ≥99.999% trace metals basis) to form a tin nitrate solution. Tin nitrate was subsequently precipitated by addition of high–purity NH_4OH. After filtration, the resulting precursor was thermally treated to obtain a dry powder. Phase purity of the final $BaSnO_3$ product was confirmed by X–ray diffraction.

The tin–containing precursor was mixed in stoichiometric proportions with $BaCO_3$ (Sigma–Aldrich, CAS 513–77–9) and thoroughly homogenized. The mixture was calcined in zirconia boat crucibles in an oxygen–vented tube furnace at 1500 K for 12 h. The resulting material was recovered and pressed into pellets with a thickness of 0.5 mm and a diameter of 8 mm using an applied force of 150 kN. No additional sintering step was performed.

*Corresponding author: artur.braun@alumni.ethz.ch

Crystallographic structure and phase purity

High-resolution neutron diffraction (ND) and anomalous X-ray diffraction (XRD) confirm that $BaSnO_3$ and $BaSn_{0.9}Y_{0.1}O_{3-\delta}$ remain single-phase cubic perovskites under dry, H_2O- and D_2O-treated conditions.

Neutron diffraction

Neutron diffraction patterns of nominally dry $BaSnO_3$ and $BaSn_{0.9}Y_{0.1}O_{3-\delta}$ confirm the cubic perovskite structure (space group $Pm\bar{3}m$) over the investigated temperature range (1–200 K), in agreement with previous reports.

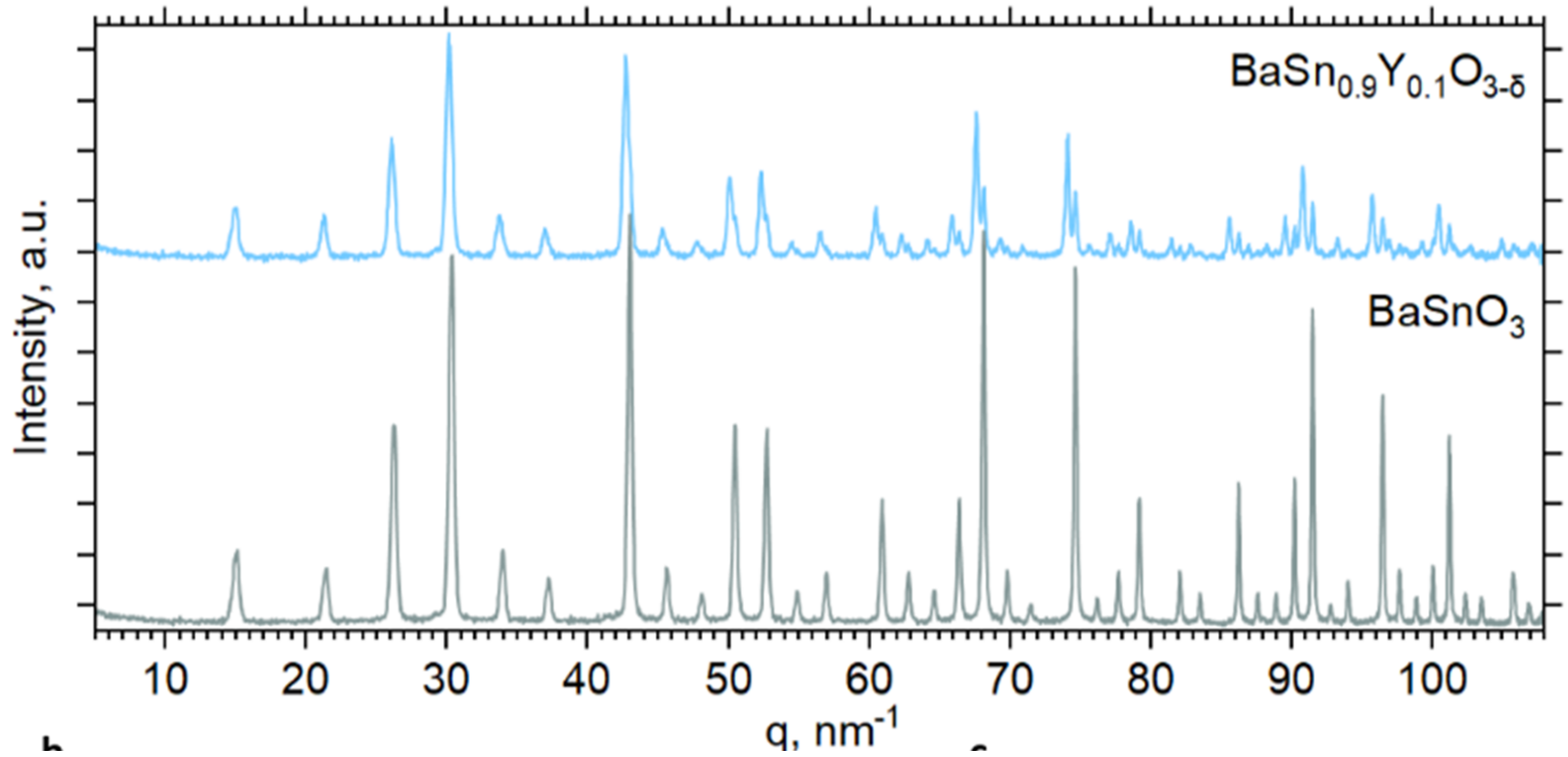

Figure S1: **Neutron diffraction and superstructure model of BSY10.** (a) Neutron diffractograms of $BaSnO_3$ (bottom, gray) and $BaSn_{0.9}Y_{0.1}O_3$ (top, blue); the patterns are vertically offset for clarity. (b) Calculated neutron diffraction pattern of $BaSn_{7/8}Y_{1/8}O_3$ compared with the experimental data; additional reflections arising from the superstructure are marked by vertical lines. The Chebyshev polynomial background has been subtracted from the experimental data. (c) Schematic representation of the $BaSn_{7/8}Y_{1/8}O_3$ supercell.

Rietveld refinements were performed with the Ba occupancy fixed to unity, while Sn and O occupancies were constrained to be identical at all temperatures. The refined values for $BaSnO_3$ correspond to Sn= 0.970(3) and O= 0.975(3), yielding a composition $BaSn_{0.97}O_{2.925}$. Charge balance considerations imply that a small fraction (∼1.5 %) of Sn is present as Sn^{2+}, corresponding to an intrinsic oxygen vacancy concentration of approximately 2.5 %. These intrinsic vacancies provide a finite population of potential proton incorporation sites even in nominally undoped $BaSnO_3$.

The refined lattice parameter of $BaSnO_3$ ($a \approx 4.11$ Å) and its temperature dependence are consistent with predominantly harmonic thermal vibrations. The isotropic displacement parameters U_{iso}(Ba,Sn) and anisotropic oxygen displacements U_{11} and U_{33} increase monotonically with temperature, with oxygen exhibiting the largest and most anisotropic motion. No additional Bragg reflections or peak splitting are observed upon hydration or deuteration, indicating that OH/OD incorporation does not induce long-range structural ordering or symmetry lowering.

Figure S1 compares neutron diffraction patterns of nominally dry $BaSnO_3$ and $BaSn_{0.9}Y_{0.1}O_{3-\delta}$. Both materials adopt the cubic perovskite structure (space group $Pm\bar{3}m$) without additional Bragg reflections or peak splitting. Rietveld refinements confirm phase purity and yield lattice parameters and displacement parameters consistent with harmonic thermal vibrations (Table S1). Full refinements are reported in Ref. [1].

Table S1: Refined structure parameters for $BaSnO_3$ at 1, 100, and 200 K.

$BaSnO_3$, wR = 5.11%			
T (K)	1	100	200
a (Å)	4.11016(7)	4.11113(10)	4.11329(11)
Ba occ.		1	
Sn occ.		0.970(3)	
O occ.		0.975(3)	
U_{iso}(Ba) (Å^2)	0.00167(12)	0.00272(17)	0.00402(18)
U_{iso}(Sn) (Å^2)	0.00068(10)	0.00120(15)	0.00184(16)
U_{11}(O) (Å^2)	0.00498(11)	0.00571(17)	0.00768(19)
U_{33}(O) (Å^2)	0.00228(21)	0.00299(31)	0.0033(3)

Anomalous (resonant) X-ray diffraction

Anomalous X-ray powder diffraction measurements were performed to probe possible cation ordering and secondary phases in $BaSnO_3$ and $BaSn_{0.9}Y_{0.1}O_{3-\delta}$. Diffraction patterns were recorded at photon energies tuned across the Sn K edge (28.8–29.8 keV) and, for BSY10, additionally across the Y K edge (16.0–18.0 keV). Instrument calibration and detector geometry were established using LaB_6 as a standard and applied consistently across all energies.

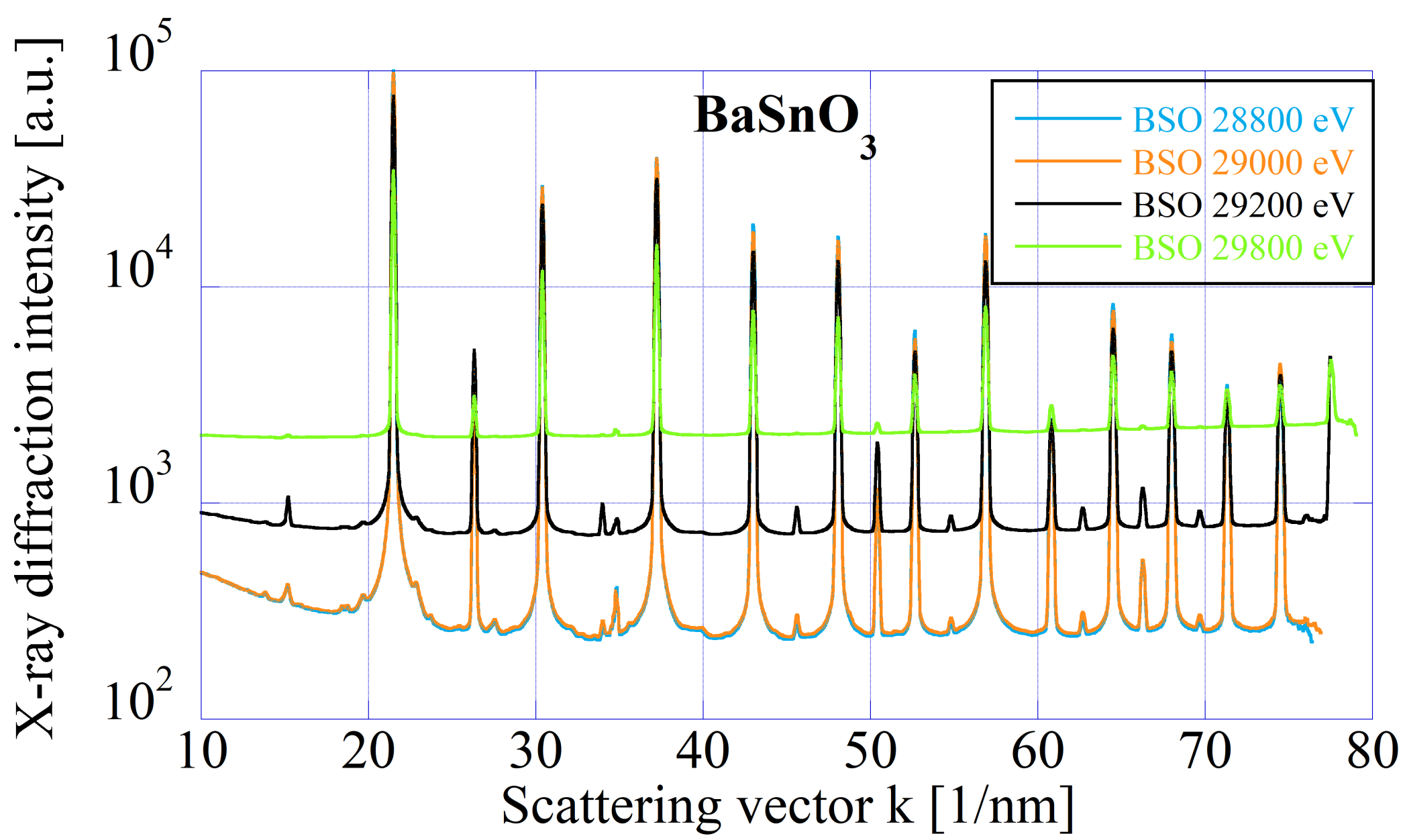

Figure S2: **Resonant X-ray diffraction of $BaSnO_3$ at the Sn edge.** X-ray diffractograms of $BaSnO_3$ recorded with photon energies between 28 800 and 29 800 eV, spanning the Sn absorption edge.

For $BaSnO_3$, anomalous diffraction across the Sn K edge (Fig. S2) reveals identical peak positions and line shapes at all photon energies, with only the expected energy-dependent intensity variations arising from anomalous dispersion. No additional reflections or peak broadening are observed, confirming that the material is single-phase and free of detectable Sn-rich secondary phases within the sensitivity of the experiment.

For the Y-doped compositions BSY10 and BSY20, tuning across the Sn and Y absorption edges leads to pronounced but smooth modulations of Bragg intensities, demonstrating that both Sn and Y contribute to the same crystallographic B-site. At all energies, the diffraction patterns exhibit sharp, symmetric reflections with identical peak positions, indicating the absence of long-range ordered superstructures or symmetry

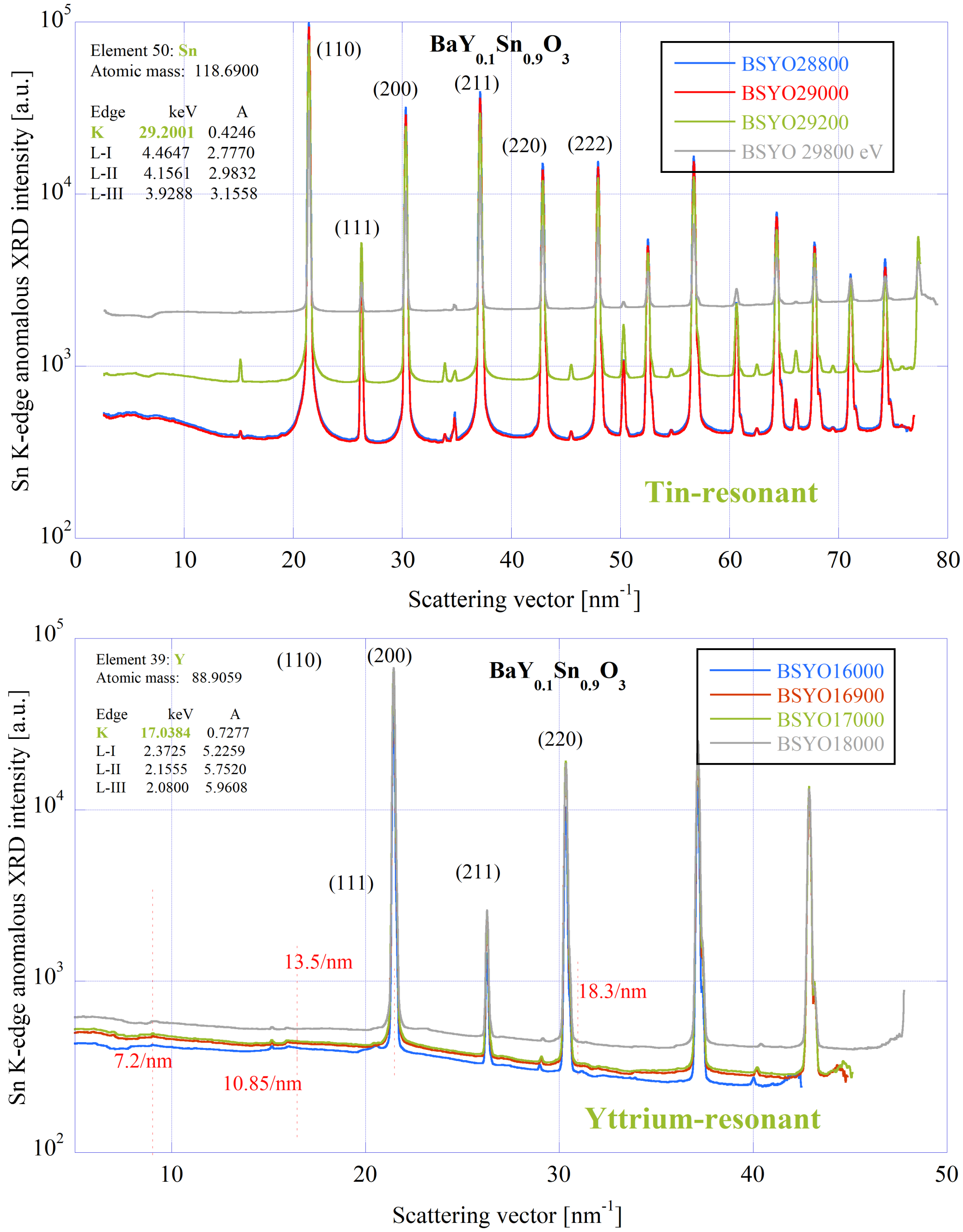

Figure S3: **Resonant X-ray diffraction of BSY10 at the Sn and Y edges.** X-ray diffractograms of $BaSn_{0.9}Y_{0.1}O_3$ recorded at eight photon energies around the Sn (top) and Y (bottom) absorption edges.

lowering.

Weak, broad energy-dependent intensity features observed at low scattering vectors in BSY10 under Y-resonant conditions (Fig. S3) point to short-range, Y-induced local distortions of the Sn–O network. These features do not correspond to commensurate superlattice peaks and are consistent with locally heterogeneous coordination environments inferred independently from XPS/UPS and vibrational spectroscopy. Overall, the anomalous XRD data confirm that Y substitution introduces local structural disorder without altering the average cubic perovskite framework.

XRD on BSY20

The lattice parameter of BSY20 measured with a laboratory X-ray diffractometer using Cu Kα radiation is 4.162 A, based on the diffractogram in Figure S3 and the Rietveld refinement with data detailed in Table S2.

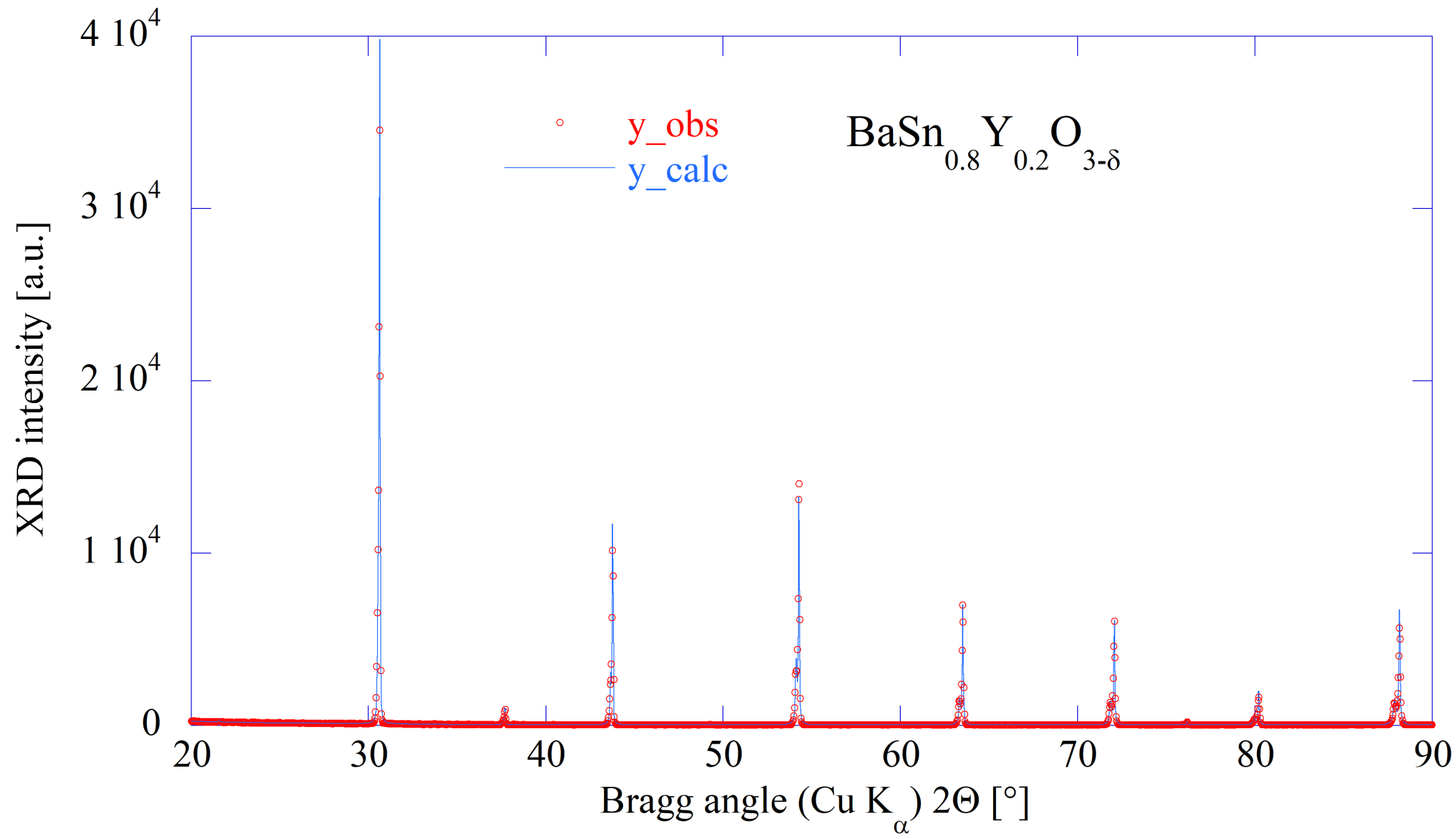

Figure S4: **XPS spectra.** High-resolution Sn 3d regions of $BaSnO_{3-\delta}$ (top) and $BaSn_{0.9}Y_{0.1}O_{3-\delta}$ (bottom). Binding energies are not corrected to the C 1s reference.

The lattice parameter of BSY20 measured with a laboratory X-ray diffractometer using Cu Kα radiation is 4.162 A. Attached is the pattern of BSY20 sample on Cu K-a.

Table S2: Refined structure parameters for BSY20 and BSY20-2 at 300K.

BSO / BSO-2, wR = 18.71%		
Parameter	BSO	BSO-2
a (Å)	4.15137(11)	4.16191(15)
b (Å)	4.15137(11)	4.16191(15)
c (Å)	4.15137(11)	4.16191(15)
Volume (Å^3)	71.544(6)	72.090(8)
Weight fraction	0.709(8)	0.291(8)
Phase fraction	—	0.444(18)
O occupancy	1.00(5)	0.73(8)
U_{iso}(Ba) (Å^2)	-0.0002(16)	—
U_{iso}(Sn) (Å^2)	-0.0040(19)	—
U_{iso}(O) (Å^2)	-0.013(7)	—
Zero offset (°)	0.275(7)	
Sample displacement (µm)	165(15)	

XPS Analysis

High-resolution Sn 3d XPS spectra reveal a clear difference between undoped $BaSnO_3$ (BSO) and Y-substituted $BaSn_{0.9}Y_{0.1}O_{3-\delta}$ (BSY10). As visible in Fig. S5,BSO exhibits a single Sn $3d_{5/2}$ component at $\sim$ 485.5 eV,

characteristic of Sn^{4+} in a regular octahedral SnO_6 environment. In contrast, BSY10 consistently shows two Sn $3d_{5/2}$ components: Sn1 at $\sim$ 485.5 eV and Sn2 at $\sim$ 486.4–486.5 eV. The higher binding-energy component reflects a locally modified Sn coordination, attributed to Y substitution and associated oxygen-vacancy compensation.

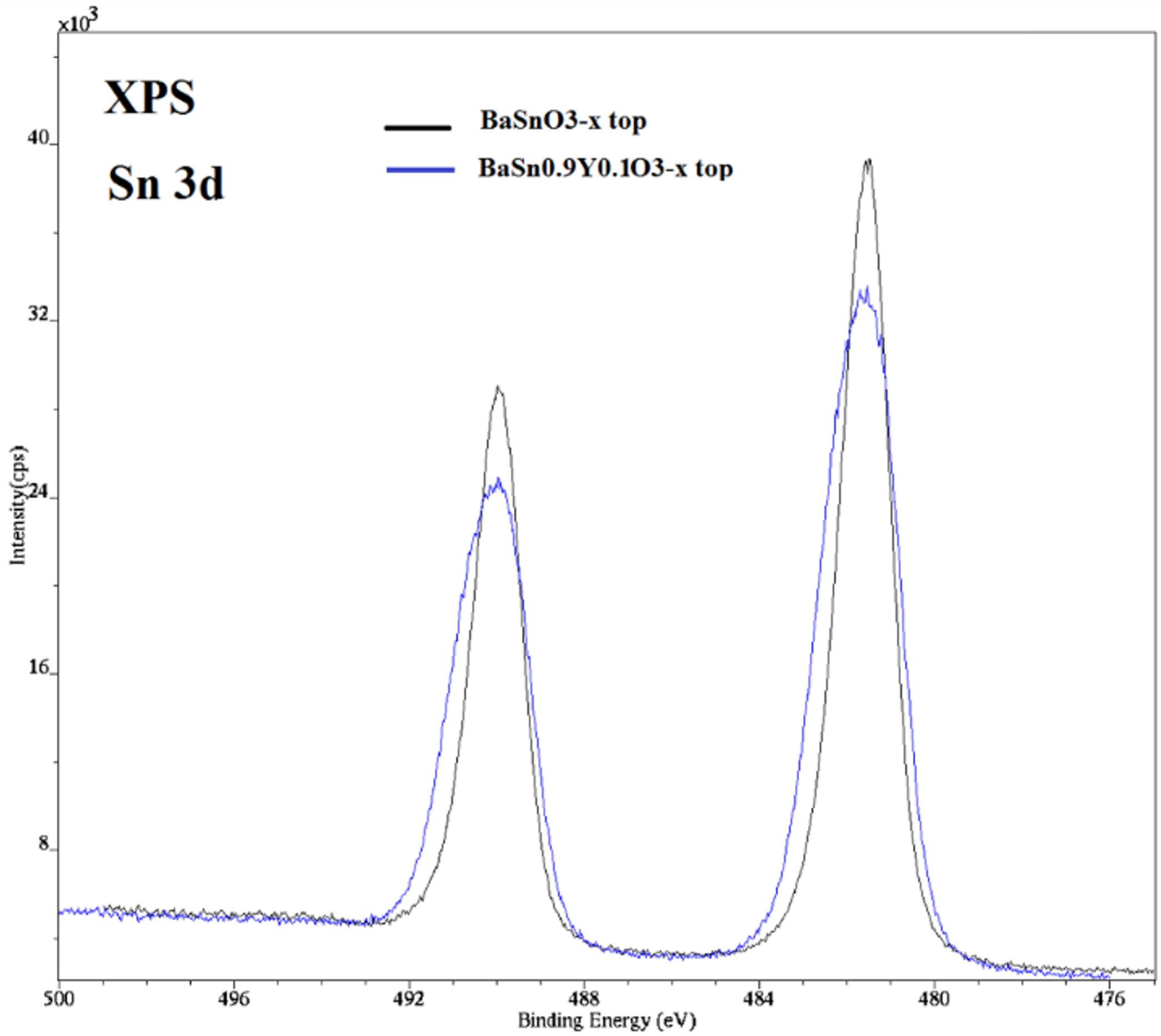

Figure S5: **XPS spectra.** High-resolution Sn 3d regions of $BaSnO_{3-\delta}$ (top) and $BaSn_{0.9}Y_{0.1}O_{3-\delta}$ (bottom). Binding energies are not corrected to the C 1s reference.

The emergence of two distinct Sn environments in BSY10 demonstrates that Y doping introduces local chemical heterogeneity while preserving the average perovskite structure. Consistently, the O 1s spectra indicate an increased contribution of lattice-stabilized hydroxyl species in BSY10 compared to BSO, providing an independent spectroscopic signature of defect-assisted hydration.

Nuclear resonance vibrational spectroscopy (NRVS)

^{119}Sn nuclear resonance vibrational spectroscopy (NRVS) was performed at 300 K. Because the nuclear resonance of ^{119}Sn is excited at $E = 23.875$ keV, the incident X-ray beam penetrates the full pellet thickness (0.5–1 mm), and the resulting spectra represent a bulk-averaged PDOS.

For each sample condition, 32 scans were co-added. All spectra were normalized to identical inelastic area in the 10–25 meV range to allow quantitative comparison between dry, H_2O-hydrated, and D_2O-hydrated samples. The instrumental energy resolution was approximately 1 meV (FWHM).

Measurements were performed sequentially under H_2O, vacuum, and D_2O atmospheres. The ^{119}Sn-projected PDOS was extracted using standard NRVS procedures as described in Ref. [2]. Spectra are shown prior to background subtraction and normalization.

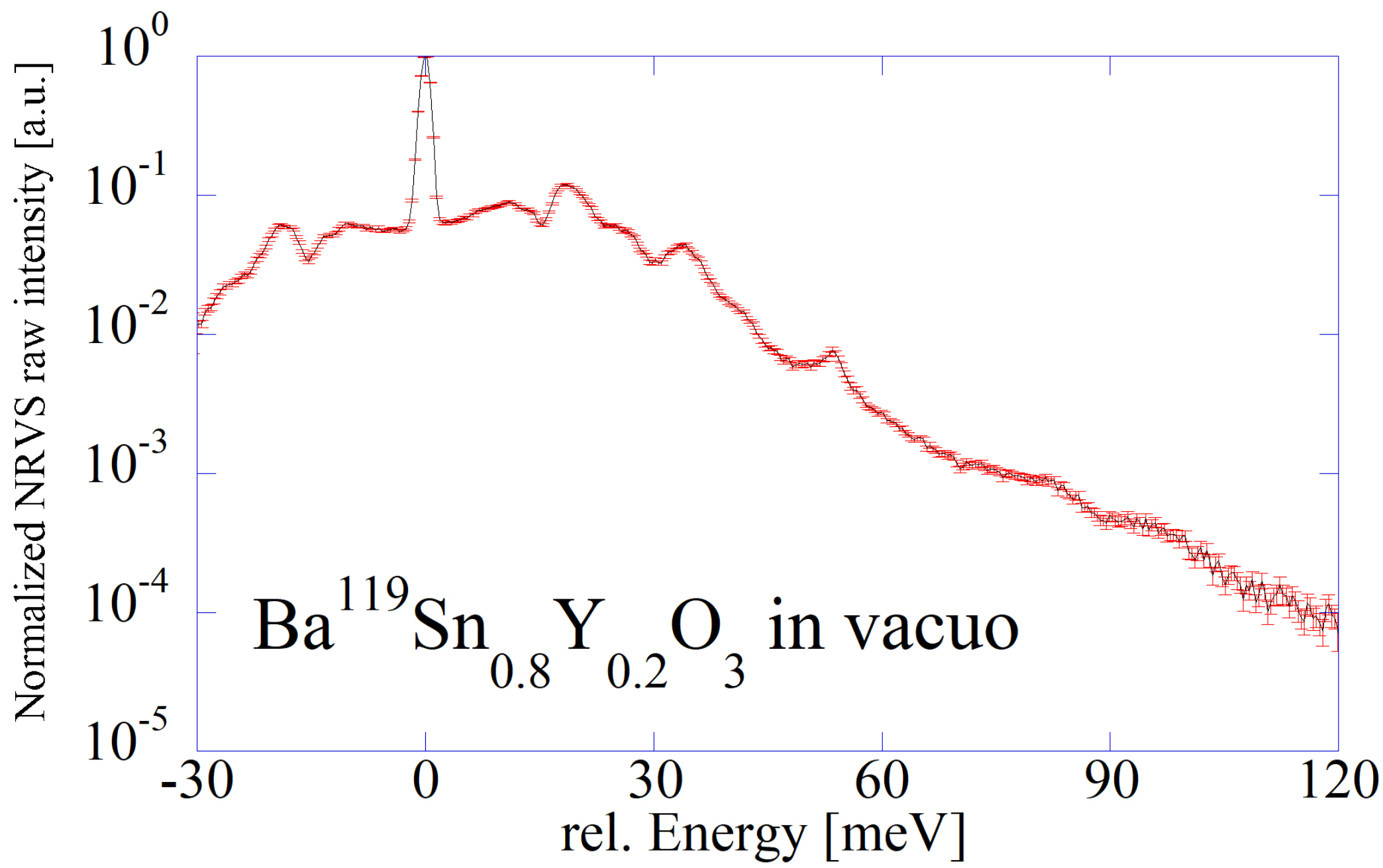

Figure S6: **Normalized NRVS spectrum of $BaSn_{0.8}Y_{0.2}O_{3-\delta}$in vacuum.** $BaSn_{0.8}Y_{0.2}O_{3-\delta}$ under vacuum-dried conditions.

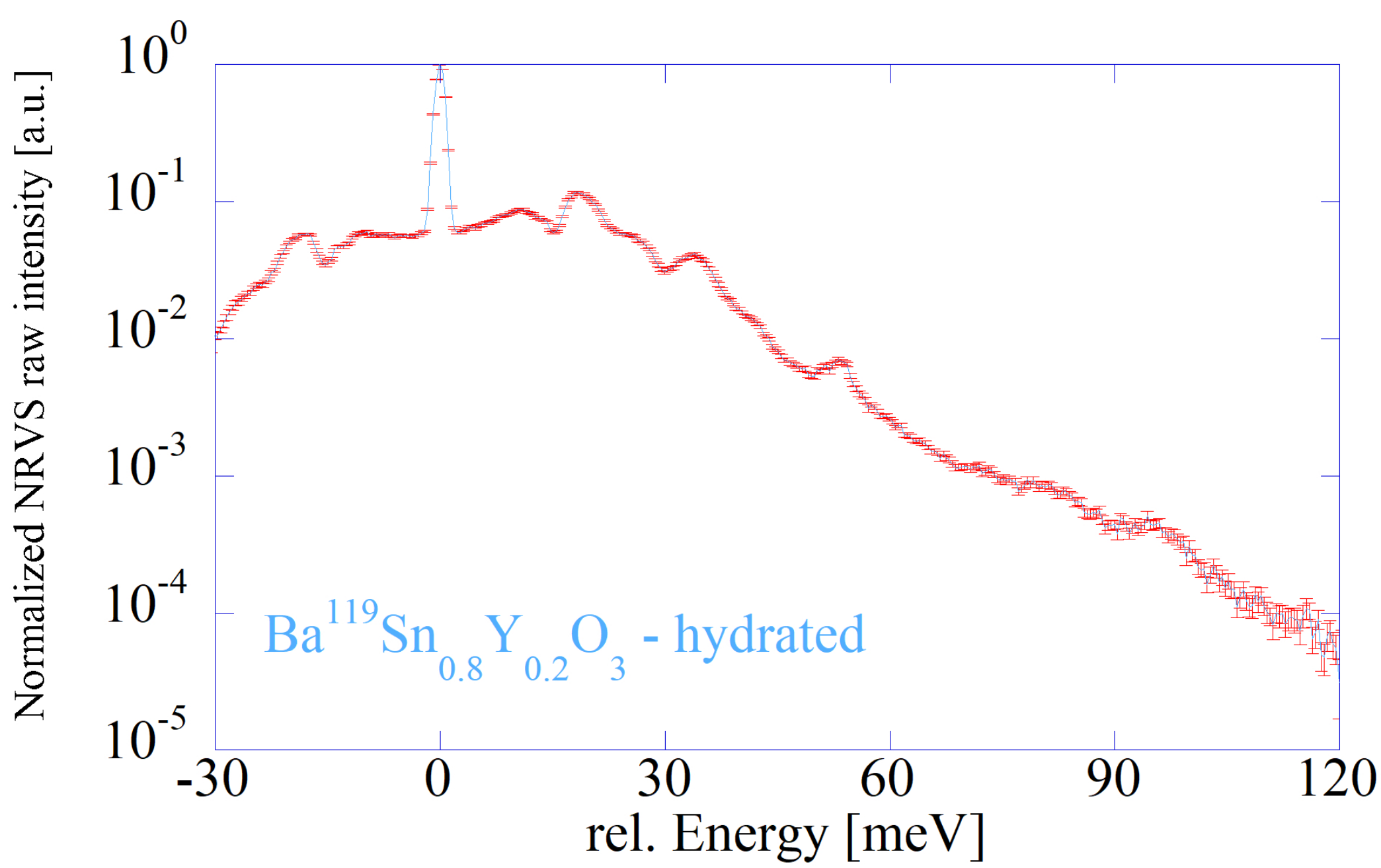

Figure S7: **Normalized NRVS spectrum of $BaSn_{0.8}Y_{0.2}O_{3-\delta}$during hydration.** $BaSn_{0.8}Y_{0.2}O_{3-\delta}$ under hydration conditions.

Figures S8 and S9 show the normalized isotope-difference spectra, $(g_{\mathrm{Sn}}^{\mathrm{D2O}} - g_{\mathrm{Sn}}^{\mathrm{H2O}})/g_{\mathrm{Sn}}^{\mathrm{dry}}$, for BSY20 and BSY10, respectively. These representations complement the absolute difference spectra shown in the main text by emphasizing relative changes across the full spectral range. They confirm that the dominant isotope-

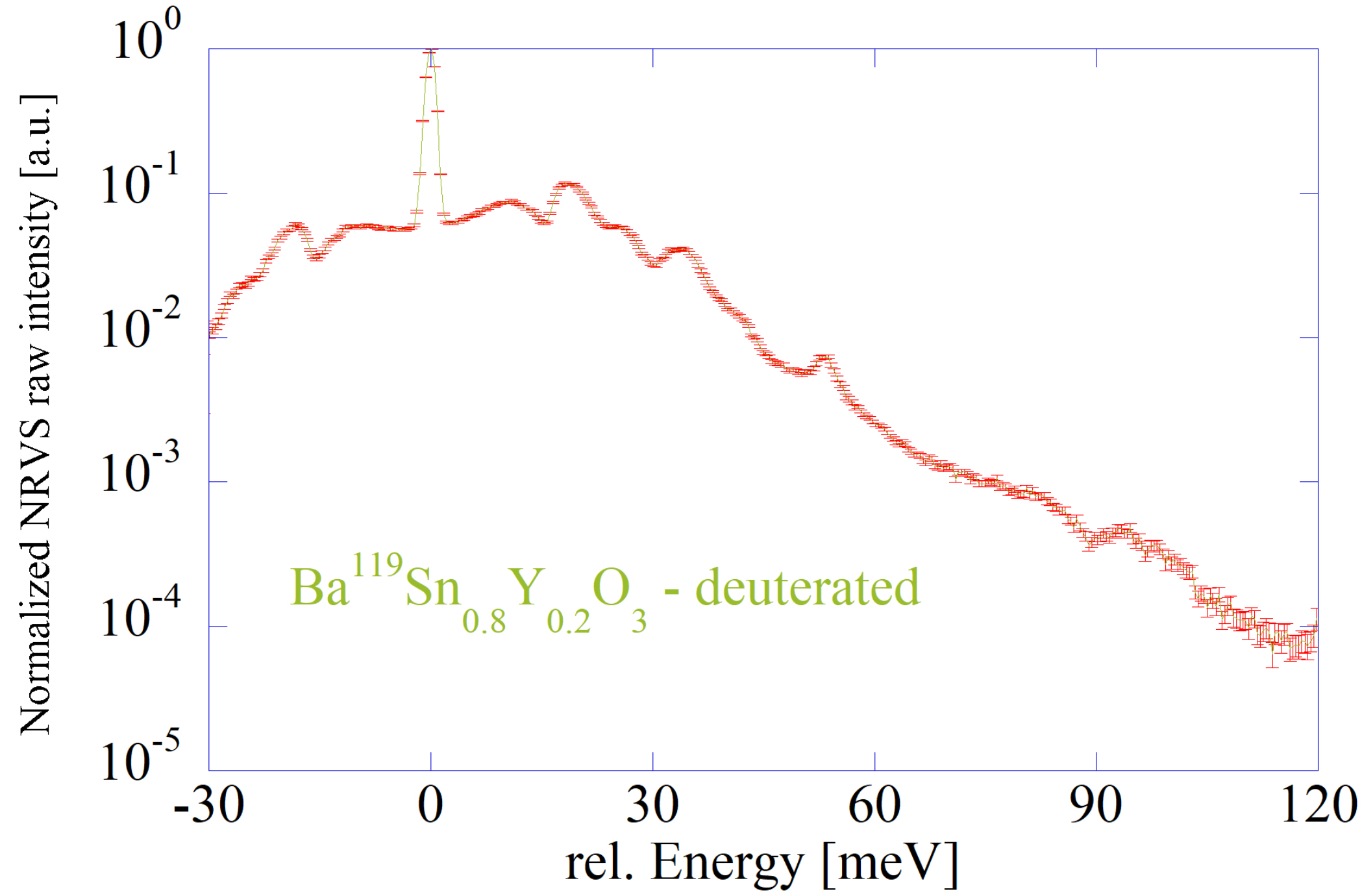

Figure S8: **Normalized NRVS spectrum of $BaSn_{0.8}Y_{0.2}O_{3-\delta}$during deuteration.** $BaSn_{0.8}Y_{0.2}O_{3-\delta}$ under deuteration conditions.

dependent response remains concentrated in the low- and mid-energy region associated with Sn–O bending and mixed lattice modes. At higher energies, where $g_{\mathrm{Sn}}^{\mathrm{dry}}$ becomes small, the normalization amplifies noise-like fluctuations and should therefore be interpreted with caution. Thus, the normalized spectra refine the visualization of relative changes but do not alter the main physical interpretation derived from the absolute difference spectra.

NRVS time-series analysis for wet BSYO

In order to assess possible beam-induced changes of the Sn-projected PDOS, we recorded a time series of 80 consecutive NRVS spectra on the same hydrated BSYO pellet ("wet BSYO") at 300 K. The incident X-ray beam size was approximately 2×10 mm^2 on the sample, and the pellet thickness was on the order of 0.5–1 mm. Thus, any dehydration caused by the beam and the vacuum environment is expected to affect only a thin surface layer on the front and back faces of the pellet, while the bulk of the volume remains essentially unaffected. All spectra were interpolated onto a common energy grid and normalized to constant inelastic area. Only weak changes are observed between early and late scans, confined to the 20–35 meV range and remaining at the level of a few percent.

Taken together, the 80-scan time series on wet BSYO shows:

1. no evidence for catastrophic radiation damage or major structural reorganization of the lattice, as the overall band integrals remain nearly constant and the main PDOS features are stable in energy;

2. small but systematic changes of the PDOS in the 20–35 meV region, which are fully consistent in sign and spectral shape with the independently observed wet→dry fingerprint.

Given the beam geometry and pellet thickness, it is reasonable to attribute these changes to mild dehydration of a thin surface layer (on the order of 10–20 μm at the vacuum interfaces), corresponding to only a few percent of the total probed volume. The NRVS signal, being bulk-sensitive, thus reports the bulk PDOS with only a minor admixture of a gradually dehydrated surface contribution.

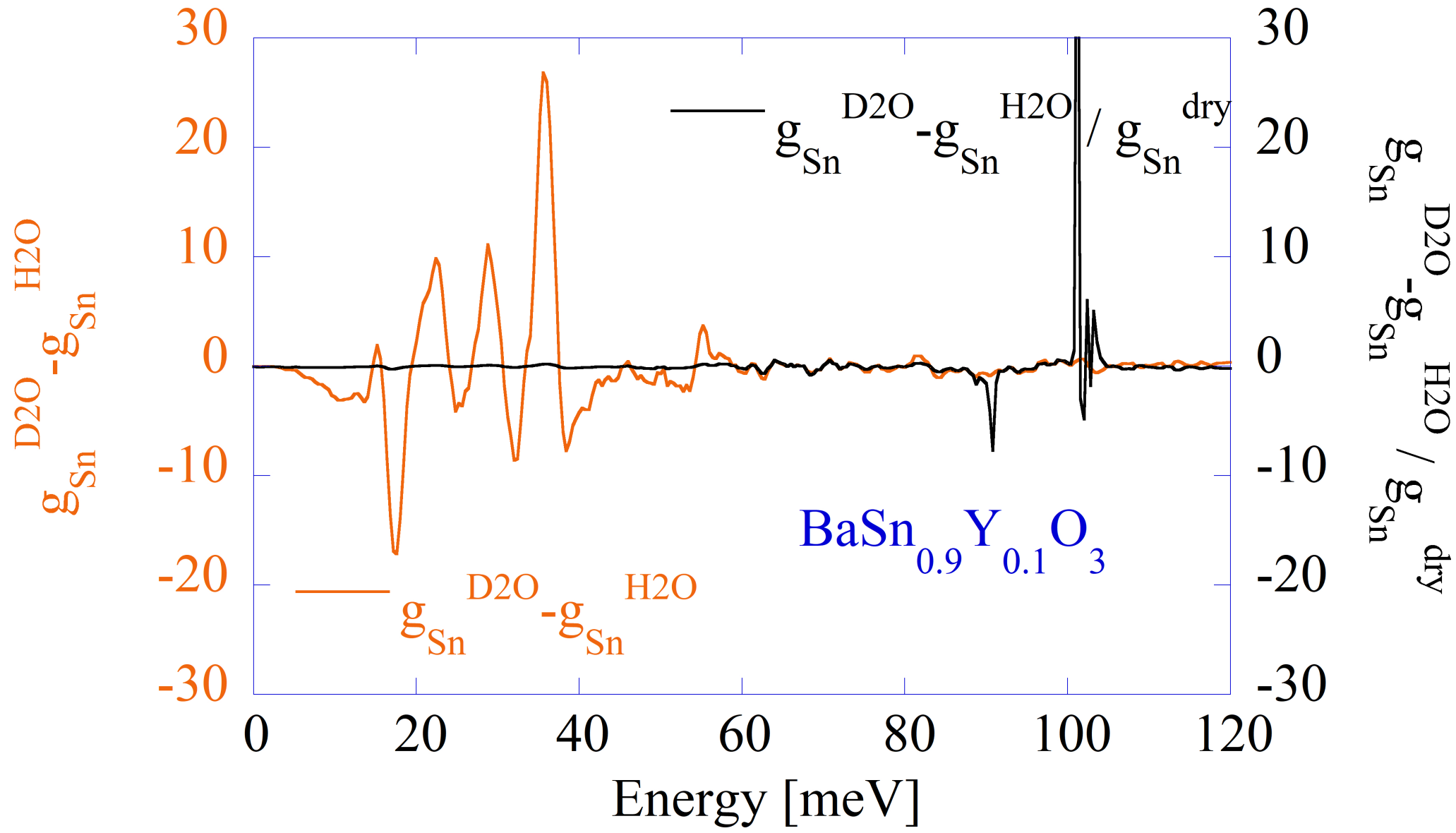

Figure S9: **Normalized isotope-difference spectrum in BSY20.** Difference spectrum $(g_{\mathrm{Sn}}^{\mathrm{D2O}} - g_{\mathrm{Sn}}^{\mathrm{H2O}})/g_{\mathrm{Sn}}^{\mathrm{dry}}$ for $\mathrm{BaSn_{0.8}Y_{0.2}O_{3-\delta}}$ (BSY20), highlighting relative isotope-sensitive spectral changes with respect to the dry sample. In spectral regions where $g_{\mathrm{Sn}}^{\mathrm{dry}}$ becomes very small, the ratio becomes noise-amplified and should not be overinterpreted.

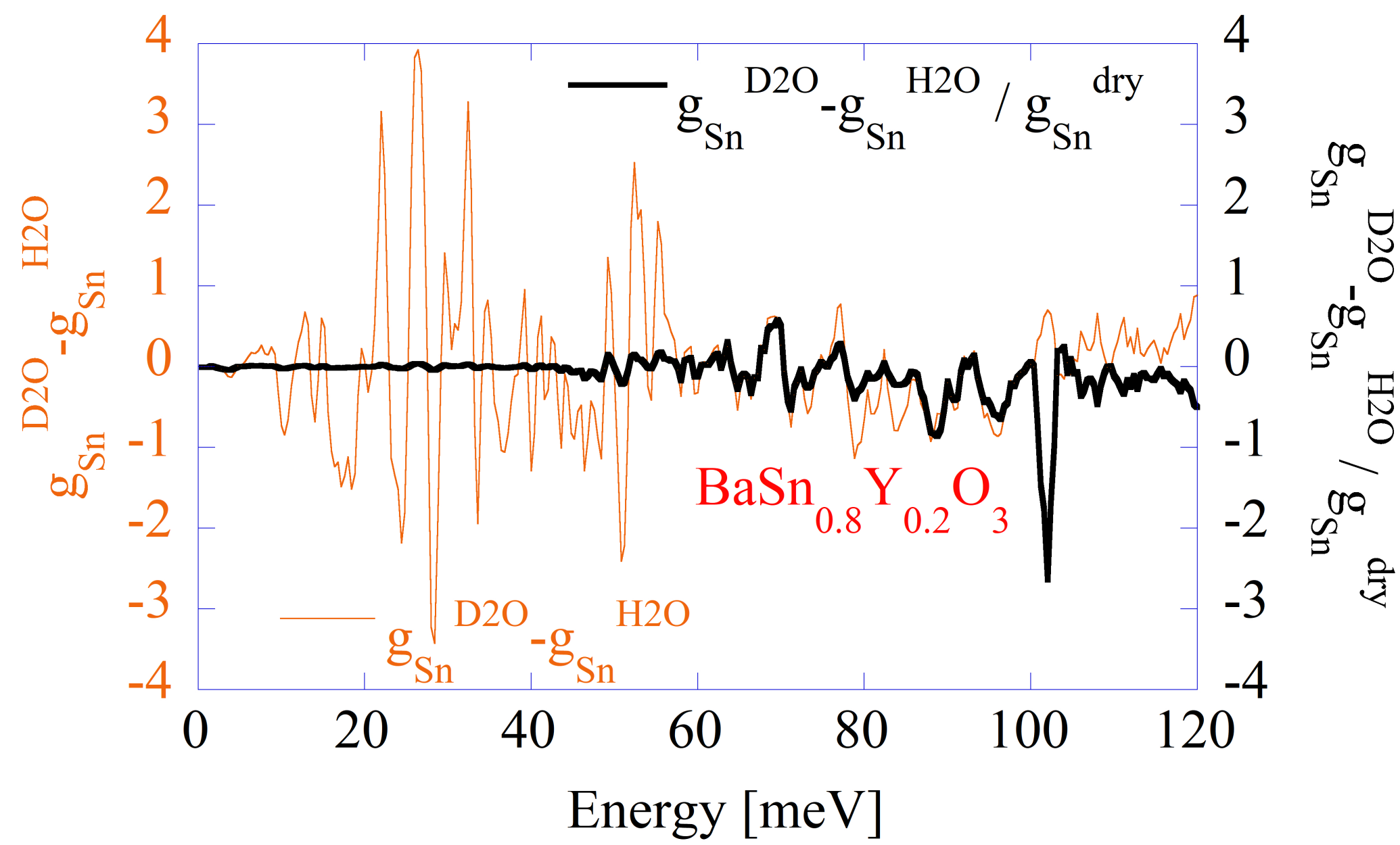

Figure S10: **Normalized isotope-difference spectrum in BSY10.** Difference spectrum $(g_{\mathrm{Sn}}^{\mathrm{D2O}} - g_{\mathrm{Sn}}^{\mathrm{H2O}})/g_{\mathrm{Sn}}^{\mathrm{dry}}$ for $\mathrm{BaSn_{0.9}Y_{0.1}O_{3-\delta}}$ (BSY10), highlighting relative isotope-sensitive spectral changes with respect to the dry sample. In spectral regions where $g_{\mathrm{Sn}}^{\mathrm{dry}}$ becomes very small, the ratio becomes noise-amplified and should not be overinterpreted.

Importantly, this internal time-series control demonstrates that the hydration-dependent PDOS features discussed in the main text (e.g. the change in peak shape around 28–35 meV between fully wet and fully dry

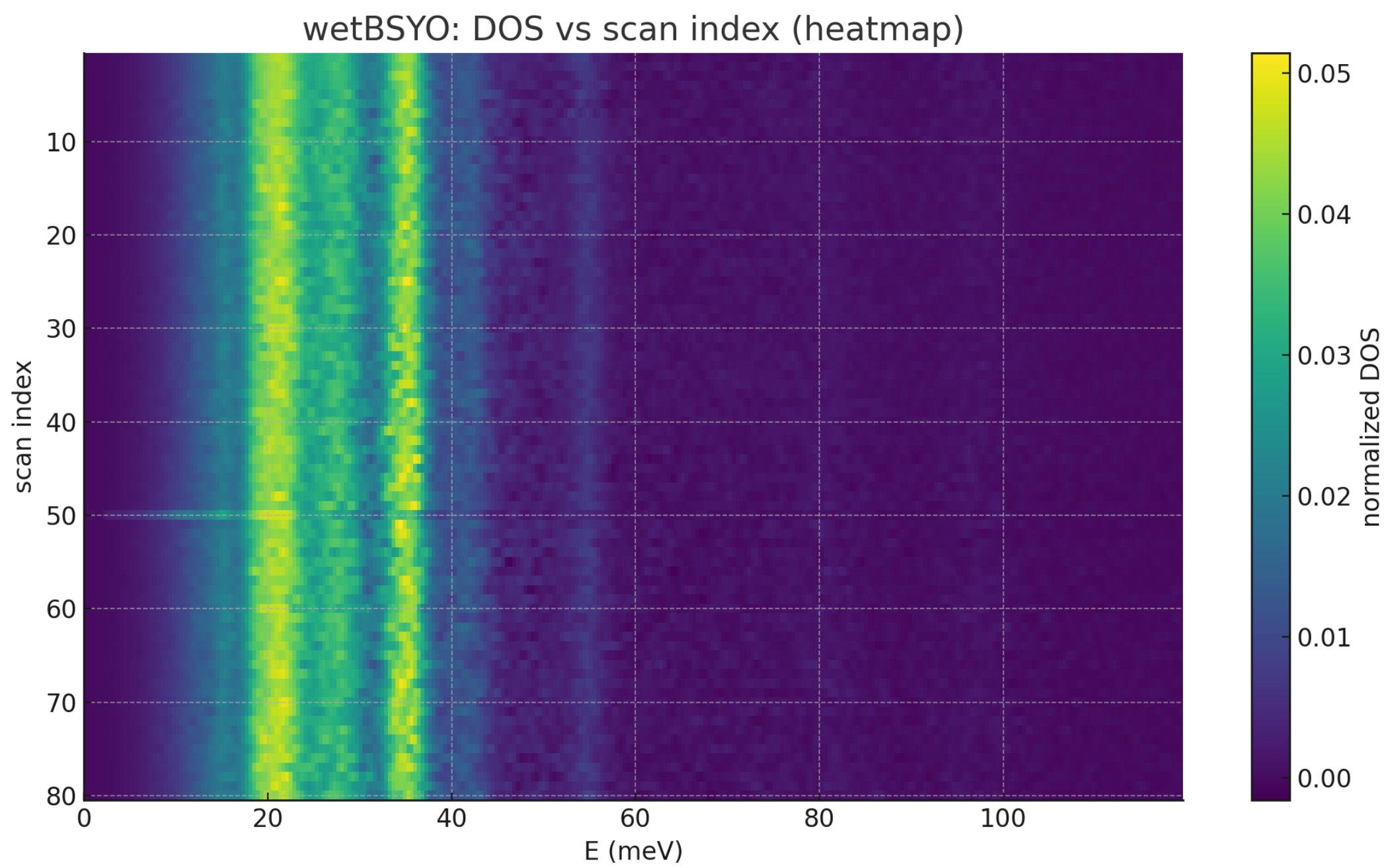

Figure S11: **Heatmap representation of the 80-scan NRVS time series for wet BSYO.** Shown is the normalized PDOS $\tilde{g}_n(E)$ for all scans $n = 1, \ldots, 80$ after interpolation onto a common energy grid. The color scale encodes PDOS intensity. All major phonon bands remain stable in energy and shape, indicating the absence of catastrophic radiation damage. Only weak intensity modulations are visible, consistent with mild surface-layer dehydration over time (see text for details).

samples) are not artifacts of sample-to-sample variability, but arise continuously and reproducibly within one and the same pellet as water is removed. The wet BSYO time series therefore strengthens, rather than weakens, the mechanistic interpretation of hydration effects on the Sn–O–Y lattice dynamics.

Estimation of the uncertainty of the first spectral moment

The first spectral moment M_1 was calculated for each individual NRVS scan prior to averaging according to

$$M_1 = \frac{\int E\, S(E)\, \mathrm{d}E}{\int S(E)\, \mathrm{d}E}.$$

This allows assessing the experimental variability of M_1 directly from the distribution of individual scans.

For each sample condition, the mean value of M_1, the standard deviation (SD), and the standard error of the mean (SEM) were determined from all full-length scans. The SEM is defined as $\mathrm{SEM} = \mathrm{SD}/\sqrt{N}$, where N is the number of scans.

Table S3: First spectral moment M_1 and associated statistical parameters obtained from individual NRVS scans for BSY20 samples.

Sample	N	M_1 (meV)	SD (meV)	SEM (meV)
BSY20 dry	38	26.55	0.27	0.04
BSY20 H_2O	38	26.85	0.28	0.05
BSY20 D_2O	38	26.92	0.26	0.04

The scan-to-scan standard deviation of M_1 is approximately 0.25–0.30 meV, while the SEM of the averaged spectra is on the order of 0.04–0.05 meV. This demonstrates that sub-meV shifts in the first spectral moment are experimentally resolvable.

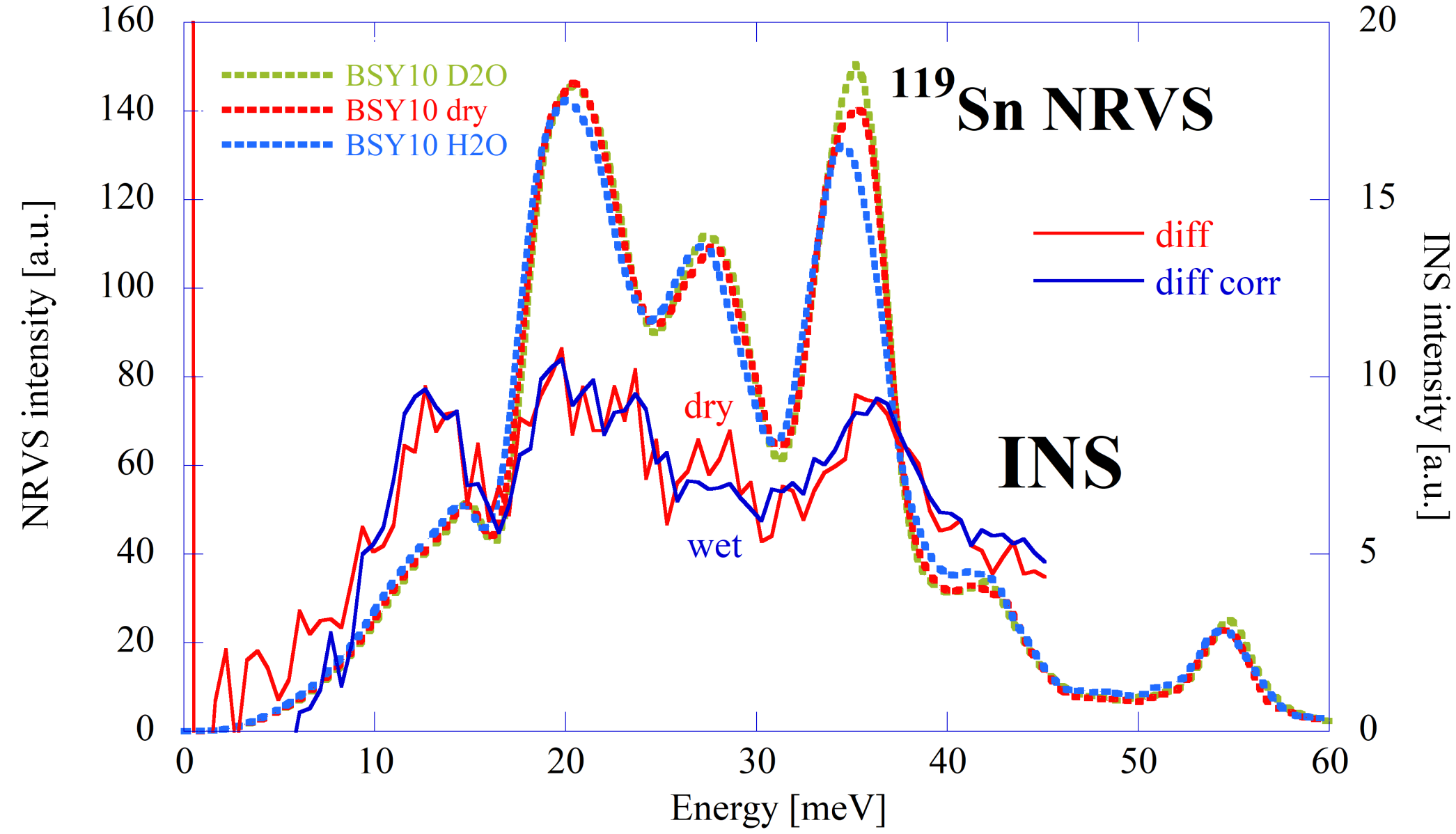

Figure S12: **Comparison of NRVS and INS.** Compared is the three PDOS $\tilde{g}_n(E)$ for BSY10 in dry and hydrated and deuterated condition at 300 K from NRVS (dotted lines), with the inealstic neutron scattering of the BSY10 in hydrated and dried condition at 723 K (solid lines, red dry, wet blue. Observe the intensities at around 27 meV.

In particular, the hydration-induced upshift (dry to H_2O) of about 0.3–0.4 meV is significantly larger than the uncertainty of the mean, confirming that the observed effect is not due to statistical fluctuations of the NRVS spectra.

Complementary INS data and qualitative mode assignment.

Inelastic neutron scattering (INS) spectra of $BaSn_{0.9}Y_{0.1}O_{3-\delta}$ (BSY10) were recorded at 723 K under nominally dry and hydrated conditions (Figure S11, left).

Samples used were ceramic pellets of the BSY material. Samples were suspended in a sealed Pt sample holder using a Pt wire in a hydrated state (hydration procedure elsewhere? Samples were heated at 200C in tube furnace with constant flow of nitrogen gas purged through bubbler with water heated to 70C). Container was sealed with ambient air and connected to a pump through a valve. Samples were measured at RT, then at elevated temperature. Then the valve on the container was opened to a vacuum pump, while sample was kept at elevated temperature, and the measurement continued after 30 minutes when no visible change in INS spectra was observed. Then samples were cooled and measured at room temperature.

The sample was not repositioned between measurements, ensuring that the observed spectral changes arise solely from hydration/dehydration. The experiment was carried out at FOCUS beamline at SINQ at Paul Scherrer Institut, Switzerland [3, 4] similar to the QENS experiment in detailed in [5].

In the dry state, a broad contribution centered around $\sim$27 meV is observed, which is significantly reduced upon hydration. At the same time, changes in the quasielastic and low-energy inelastic background are apparent, consistent with increased proton-related dynamics in the hydrated state.

For qualitative interpretation, calculated partial vibrational densities of states (pDOS) for $BaSnO_3$ are shown in Figure S11 (right). These calculations indicate that the 20–35 meV energy range corresponds predominantly to mixed Sn–O and O-dominated lattice modes, rather than to localized proton vibrations.

We therefore attribute the hydration-dependent reduction of the $\sim$27 meV feature to the removal of oxygen-vacancy-related lattice modes associated with locally under-coordinated environments. Upon hydration, filling of oxygen vacancies and formation of hydroxyl groups eliminates these softer configurations and leads to a redistribution of spectral weight within the collective lattice modes.

These INS results are fully consistent with the NRVS observations, which show that hydration modifies the Sn-projected phonon density of states primarily through changes in the local force-constant landscape, while proton-related vibrations remain largely decoupled from the Sn-weighted spectral response.

The INS feature observed around ∼27 meV coincides with the energy range identified by NRVS as dominated by Sn–O bending and mixed lattice modes. This correspondence indicates that the hydration-dependent changes observed by INS originate from modifications within an existing collective vibrational band, rather than from the emergence of distinct proton-localized modes.

References

[1] A. Rulev, N. Nagasawa, H. Wang, V. Pomjakushin, M. Kunz, Y. Yoda, S. P. Cramer, Q. Chen, A. Braun, *Adv Sci (Weinh)* **2025**, e07261.

[2] A. Rulev, H. X. Wang, S. Erat, M. Aycibin, D. Rentsch, V. Pomjakushin, S. P. Cramer, Q. L. Chen, N. Nagasawa, Y. Yoda, A. Braun, *Crystals* **2025**, *15*, 5.

[3] S. Janssen, F. Altorfer, L. Holitzner, R. Hempelmann, *Physica B* **2000**, *276* 89–90.

[4] C. Beck, S. Janssen, B. Gross, R. Hempelmann, *Scripta Materialia* **2001**, *44*, 8-9 2309–2313.

[5] A. Braun, S. Duval, P. Ried, J. Embs, F. Juranyi, T. Strässle, U. Stimming, R. Hempelmann, P. Holtappels, T. Graule, *Journal of Applied Electrochemistry* **2009**, *39*, 4 471–475.
